# Inference of miRNA targets using evolutionary conservation and pathway analysis

**DOI:** 10.1186/1471-2105-8-69

**Published:** 2007-03-01

**Authors:** Dimos Gaidatzis, Erik van Nimwegen, Jean Hausser, Mihaela Zavolan

**Affiliations:** 1 Biozentrum, University of Basel, Basel, Switzerland; 2 Swiss Institute of Bioinformatics, Basel, Switzerland

## Abstract

**Background:**

MicroRNAs have emerged as important regulatory genes in a variety of cellular processes and, in recent years, hundreds of such genes have been discovered in animals. In contrast, functional annotations are available only for a very small fraction of these miRNAs, and even in these cases only partially.

**Results:**

We developed a general Bayesian method for the inference of miRNA target sites, in which, for each miRNA, we explicitly model the evolution of orthologous target sites in a set of related species. Using this method we predict target sites for all known miRNAs in flies, worms, fish, and mammals. By comparing our predictions in fly with a reference set of experimentally tested miRNA-mRNA interactions we show that our general method performs at least as well as the most accurate methods available to date, including ones specifically tailored for target prediction in fly. An important novel feature of our model is that it explicitly infers the phylogenetic distribution of functional target sites, independently for each miRNA. This allows us to infer species-specific and clade-specific miRNA targeting. We also show that, in long human 3' UTRs, miRNA target sites occur preferentially near the start and near the end of the 3' UTR.

To characterize miRNA function beyond the predicted lists of targets we further present a method to infer significant associations between the sets of targets predicted for individual miRNAs and specific biochemical pathways, in particular those of the KEGG pathway database. We show that this approach retrieves several known functional miRNA-mRNA associations, and predicts novel functions for known miRNAs in cell growth and in development.

**Conclusion:**

We have presented a Bayesian target prediction algorithm without any tunable parameters, that can be applied to sequences from any clade of species. The algorithm automatically infers the phylogenetic distribution of functional sites for each miRNA, and assigns a posterior probability to each putative target site. The results presented here indicate that our general method achieves very good performance in predicting miRNA target sites, providing at the same time insights into the evolution of target sites for individual miRNAs. Moreover, by combining our predictions with pathway analysis, we propose functions of specific miRNAs in nervous system development, inter-cellular communication and cell growth. The complete target site predictions as well as the miRNA/pathway associations are accessible on the ElMMo web server.

## Background

Since the initial discovery of the lin-4 miRNA [1], and then of the let-7 miRNA which is highly conserved in evolution [2], combined experimental and computational approaches have resulted in the identification of hundreds of miRNAs in animal genomes, some of the large-scale studies being [3-19]. In contrast, high-throughput approaches for experimental identification of miRNA *targets *are only in their infancy [20,21], and global properties of miRNA-dependent regulatory networks have mostly been inferred from computationally-predicted target sites [22-26].

Perhaps surprisingly, relatively little is known about the constraints on a functional miRNA target site. Mutational studies [27,28] confirmed initial observations of Lai [29] and Lewis et al. [30] that perfect base pairing between the 5' end of the miRNA and its target is essential. As a consequence, some of the computational methods for miRNA target prediction require [22,31] or can enforce the constraint [32] that 6–8 nucleotides at the 5' end of the miRNA, the so-called miRNA "seed", are perfectly base paired with its mRNA target, or give a higher weight to the base pairs formed in this region [25,33]. Since every 6mer occurs on average once every 4,096 nucleotides in random sequence, the number of target sites for each miRNA would be very large if matching of the seed were the only requirement for functional target sites. Although there are indications that miRNAs do have a large number of targets [20-22,28,31,34], experimental studies typically do not confirm that every seed match constitutes a functional target site. It seems therefore that additional factors contribute to the functionality of target sites. To improve the specificity of prediction of functional target sites, most computational studies make use of evolutionarily conservation [22,25,31,35] or at least flag conserved putative targets [32,33]. However, currently available methods generally use conservation statistics in an *ad hoc *manner. In particular, existing methods do not explicitly take the phylogenetic relationships into account when weighing the evidence of conservation between related species. In addition, current methods treat all miRNAs identically and ignore that the selection pressures for conserving functional target sites between related species may differ significantly between miRNAs. That is, functional target sites for one miRNA may be preferentially conserved in one subset of species, whereas the functional sites for another miRNA may be preferentially conserved in another subset of species. Incorporating conservation statistics in a general, rigorous and miRNA-dependent manner are the main features of the miRNA target prediction method that we present here.

From the very early stages of miRNA target prediction it became clear that regulatory proteins such as transcription factors are preferentially subjected to miRNA-dependent regulation. Yet, beyond a few well-characterized miRNA-target interactions, there is still very little known about the place of individual miRNAs in the regulatory networks of cells and organs. Several groups [23,30,36] have used Gene Ontology categories in an attempt to characterize the biological roles of different miRNAs. Here we present a new analysis based on the association of targets for individual miRNAs with molecular pathways annotated in the KEGG database. This approach recovers some of the known miRNA-mRNA associations, and makes new predictions, in particular it predicts the for the involvement of specific miRNAs in nervous system development, inter-cellular communication, and cell growth.

## Results and Discussion

### miRNA-target interactions: the importance of different 'seed types'

**Figure 1:**
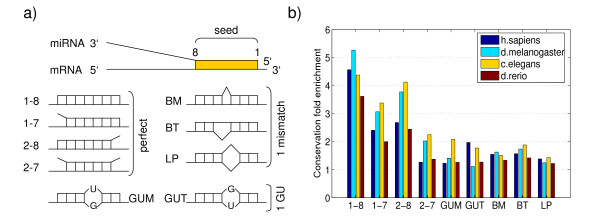
**MiRNA seed types and conservation fold enrichment**. Schematic representation of the different "seed types" of miRNA target sites that we consider and conservation fold enrichment for each of them. a. Seed type interactions of miRNA-mRNA hybrids (see text). b. Conservation fold enrichment for the 9 different seed types in the four clades.

Several lines of evidence [22,27-29,37] suggest that complementarity of the target site to the first 8 bases at the 5' end of the miRNA are of crucial importance for target site recognition. Lewis et al. [22] have investigated the importance of the miRNA "seed", defined as the positions 2–7 of the miRNA, by comparing conservation statistics of mRNA segments that are complementary to miRNA seeds with those of randomized control sets. They concluded that conserved 3' UTR regions predicted to hybridize perfectly with positions 2–8 of the miRNA or with positions 2–7 of the miRNA, but having an A nucleotide flanking the seed match at the 3' end are likely to be miRNA target sites. Inspired by these methods we decided to re-investigate the conservation statistics of different "seed types" across different clades of organisms. In all cases, we only focused on the first 8 positions of the miRNA, and we analyzed the following 9 "seed types" (see Figure 1):

1. Perfect complementarity with Watson-Crick interactions between positions 1–8 of the miRNA and the mRNA target site.

2. Perfect complementarity at positions 1–7 but not at position 8.

3. Perfect complementarity at positions 2–8 but not at position 1.

4. Perfect complementarity at 2–7 but not at 1 and 8.

5. Complementarity at positions 1–8 with a single G-U pair occurring with the U in the miRNA (GUM).

6. Complementarity at positions 1–8 with a single G-U pair occurring with the U in the target (GUT).

7. Complementarity with a single bulged nucleotide on the miRNA side (BM).

8. Complementarity with a single bulged nucleotide on the target side (BT).

9. Complementarity with a single internal loop involving one nucleotide in both miRNA and target (LP).

For each of these 9 seed types *t*, and each of the four clades (mammals plus chicken, fishes, flies, and worms), we determined the fraction *f*_*t *_of putative target sites that are perfectly conserved in all species of the clade (see Methods for details). We only considered miRNAs that were themselves conserved in all species of the clade. We also determined the "background" conservation fraction, of randomly chosen 3' UTR sequence segments of the same length as the respective seed types that are conserved in all species of the clade. Figure 1 shows the ratio of these two fractions, which we called "conservation fold enrichment". As expected, octameric sites show most evidence of functionality. The conservation fold enrichment decreases dramatically as the extent of complementarity that we require between miRNA and putative target site decreases. In particular, sites in which only the nucleotides 2–7 of the miRNA are predicted to form base pairs with the mRNA, as well as sites predicted to form G-U base pairs or to contain internal loops show relative little evidence of conservation enrichment. This is not to say that such sites are never functional. Indeed, functional target sites of this type are known, in particular in worms [38], for which, interestingly, we observe the strongest evidence of selection on these seed types. However, for our target prediction method we decided to focus on the three seed types that show strong evidence of conservation enrichment across all clades: those with perfect Watson-Crick complementarity with positions 1–7, 2–8, or 1–8 of the miRNA. As a result, we predict the same set of target sites for different miRNAs with the same first 8 nucleotides. In reality, even though the seed is probably most important for targeting, the 3' ends may also contribute to the target selection and this could differentiate the target sets for different miRNAs with the same seed. This possibility, which has been studied experimentally by Brennecke et al. [28], and is explicitly incorporated in other target prediction models [25], is not captured by our model. Note, however, that because the miRNA-mediated targeting depends on the expression of both miRNA and targets, distinct miRNAs that have the same seed sequence may still have different target sets simply due to differences in their expression profile, even though in principle they recognize the same set of target sites. Note also that we do not use a model in which the mRNA position corresponding to the first nucleotide in miRNA is an adenosine, because we did not find this constraint to consistently improve the conservation fold enrichment across all clades (not shown).

### Bayesian phylogenetic model for miRNA target sites

We have developed a Bayesian probabilistic model for assigning, to each putative "site" in a 3' UTR that is complementary to a miRNA seed, a posterior probability that the site is a functional target site for the miRNA, meaning that the site has been selected in evolution for its ability to bind the miRNA. The details of the model are described in the Methods section, but the main ingredients are the following.

For each miRNA and each seed type *t *we collect all putative sites in the 3' UTRs of the reference species of the clade in question, i.e. the 3' UTR sequence segments that are complementary to the given miRNA seed. For each of these putative sites we then determine the conservation pattern c→
 MathType@MTEF@5@5@+=feaafiart1ev1aaatCvAUfKttLearuWrP9MDH5MBPbIqV92AaeXatLxBI9gBaebbnrfifHhDYfgasaacH8akY=wiFfYdH8Gipec8Eeeu0xXdbba9frFj0=OqFfea0dXdd9vqai=hGuQ8kuc9pgc9s8qqaq=dirpe0xb9q8qiLsFr0=vr0=vr0dc8meaabaqaciaacaGaaeqabaqabeGadaaakeaacuWGJbWygaWcaaaa@2E0D@, defined as a binary vector with *c*_*i *_= 1 if the site is conserved in species *i *and *c*_*i *_= 0 if it is not conserved in species *i*. We then count the number of times *n*(c→
 MathType@MTEF@5@5@+=feaafiart1ev1aaatCvAUfKttLearuWrP9MDH5MBPbIqV92AaeXatLxBI9gBaebbnrfifHhDYfgasaacH8akY=wiFfYdH8Gipec8Eeeu0xXdbba9frFj0=OqFfea0dXdd9vqai=hGuQ8kuc9pgc9s8qqaq=dirpe0xb9q8qiLsFr0=vr0=vr0dc8meaabaqaciaacaGaaeqabaqabeGadaaakeaacuWGJbWygaWcaaaa@2E0D@, *t*) that conservation pattern c→
 MathType@MTEF@5@5@+=feaafiart1ev1aaatCvAUfKttLearuWrP9MDH5MBPbIqV92AaeXatLxBI9gBaebbnrfifHhDYfgasaacH8akY=wiFfYdH8Gipec8Eeeu0xXdbba9frFj0=OqFfea0dXdd9vqai=hGuQ8kuc9pgc9s8qqaq=dirpe0xb9q8qiLsFr0=vr0=vr0dc8meaabaqaciaacaGaaeqabaqabeGadaaakeaacuWGJbWygaWcaaaa@2E0D@ is observed for putative target sites of seed type *t*. To compute the posterior probabilities for individual sites, the model then compares these numbers *n*(c→
 MathType@MTEF@5@5@+=feaafiart1ev1aaatCvAUfKttLearuWrP9MDH5MBPbIqV92AaeXatLxBI9gBaebbnrfifHhDYfgasaacH8akY=wiFfYdH8Gipec8Eeeu0xXdbba9frFj0=OqFfea0dXdd9vqai=hGuQ8kuc9pgc9s8qqaq=dirpe0xb9q8qiLsFr0=vr0=vr0dc8meaabaqaciaacaGaaeqabaqabeGadaaakeaacuWGJbWygaWcaaaa@2E0D@, *t*) with those that would be expected given the "background" frequencies *p*(c→
 MathType@MTEF@5@5@+=feaafiart1ev1aaatCvAUfKttLearuWrP9MDH5MBPbIqV92AaeXatLxBI9gBaebbnrfifHhDYfgasaacH8akY=wiFfYdH8Gipec8Eeeu0xXdbba9frFj0=OqFfea0dXdd9vqai=hGuQ8kuc9pgc9s8qqaq=dirpe0xb9q8qiLsFr0=vr0=vr0dc8meaabaqaciaacaGaaeqabaqabeGadaaakeaacuWGJbWygaWcaaaa@2E0D@|*t*, bg) with which randomly chosen 3' UTR sequence segments of the same length as the miRNA seed show conservation pattern c→
 MathType@MTEF@5@5@+=feaafiart1ev1aaatCvAUfKttLearuWrP9MDH5MBPbIqV92AaeXatLxBI9gBaebbnrfifHhDYfgasaacH8akY=wiFfYdH8Gipec8Eeeu0xXdbba9frFj0=OqFfea0dXdd9vqai=hGuQ8kuc9pgc9s8qqaq=dirpe0xb9q8qiLsFr0=vr0=vr0dc8meaabaqaciaacaGaaeqabaqabeGadaaakeaacuWGJbWygaWcaaaa@2E0D@.

Generally, if conservation patterns with many *c*_*i *_= 1 are much more abundant among putative miRNA sites than among background sites, then we infer that a fraction of the putative target sites must be functional and that selection has maintained these sites in some of the species. However, conserved target sites need not be functional in all species in which they occur. The conservation pattern of a given site is typically the result of selection maintaining the site in some of the species in combination with chance conservation of the site in other species, in particular those that are evolutionarily close. Our model flexibly and explicitly takes this into account. The model considers all possible "selection patterns" s→
 MathType@MTEF@5@5@+=feaafiart1ev1aaatCvAUfKttLearuWrP9MDH5MBPbIqV92AaeXatLxBI9gBaebbnrfifHhDYfgasaacH8akY=wiFfYdH8Gipec8Eeeu0xXdbba9frFj0=OqFfea0dXdd9vqai=hGuQ8kuc9pgc9s8qqaq=dirpe0xb9q8qiLsFr0=vr0=vr0dc8meaabaqaciaacaGaaeqabaqabeGadaaakeaacuWGZbWCgaWcaaaa@2E2D@, which are also binary vectors, with *s*_*i *_= 1 if the site is under selection in species *i*, and *s*_*i *_= 0 if it is not.

For each miRNA we then determine the frequencies *p*(s→
 MathType@MTEF@5@5@+=feaafiart1ev1aaatCvAUfKttLearuWrP9MDH5MBPbIqV92AaeXatLxBI9gBaebbnrfifHhDYfgasaacH8akY=wiFfYdH8Gipec8Eeeu0xXdbba9frFj0=OqFfea0dXdd9vqai=hGuQ8kuc9pgc9s8qqaq=dirpe0xb9q8qiLsFr0=vr0=vr0dc8meaabaqaciaacaGaaeqabaqabeGadaaakeaacuWGZbWCgaWcaaaa@2E2D@) of different selection patterns that maximize the overall likelihood of the observed counts *n*(c→
 MathType@MTEF@5@5@+=feaafiart1ev1aaatCvAUfKttLearuWrP9MDH5MBPbIqV92AaeXatLxBI9gBaebbnrfifHhDYfgasaacH8akY=wiFfYdH8Gipec8Eeeu0xXdbba9frFj0=OqFfea0dXdd9vqai=hGuQ8kuc9pgc9s8qqaq=dirpe0xb9q8qiLsFr0=vr0=vr0dc8meaabaqaciaacaGaaeqabaqabeGadaaakeaacuWGJbWygaWcaaaa@2E0D@, *t*). That is, we determine the distribution of selection patterns *p*(s→
 MathType@MTEF@5@5@+=feaafiart1ev1aaatCvAUfKttLearuWrP9MDH5MBPbIqV92AaeXatLxBI9gBaebbnrfifHhDYfgasaacH8akY=wiFfYdH8Gipec8Eeeu0xXdbba9frFj0=OqFfea0dXdd9vqai=hGuQ8kuc9pgc9s8qqaq=dirpe0xb9q8qiLsFr0=vr0=vr0dc8meaabaqaciaacaGaaeqabaqabeGadaaakeaacuWGZbWCgaWcaaaa@2E2D@) that best explains the observed counts *n*(c→
 MathType@MTEF@5@5@+=feaafiart1ev1aaatCvAUfKttLearuWrP9MDH5MBPbIqV92AaeXatLxBI9gBaebbnrfifHhDYfgasaacH8akY=wiFfYdH8Gipec8Eeeu0xXdbba9frFj0=OqFfea0dXdd9vqai=hGuQ8kuc9pgc9s8qqaq=dirpe0xb9q8qiLsFr0=vr0=vr0dc8meaabaqaciaacaGaaeqabaqabeGadaaakeaacuWGJbWygaWcaaaa@2E0D@, *t*) of conservation patterns for this miRNA.

Using the estimated frequencies *p*(s→
 MathType@MTEF@5@5@+=feaafiart1ev1aaatCvAUfKttLearuWrP9MDH5MBPbIqV92AaeXatLxBI9gBaebbnrfifHhDYfgasaacH8akY=wiFfYdH8Gipec8Eeeu0xXdbba9frFj0=OqFfea0dXdd9vqai=hGuQ8kuc9pgc9s8qqaq=dirpe0xb9q8qiLsFr0=vr0=vr0dc8meaabaqaciaacaGaaeqabaqabeGadaaakeaacuWGZbWCgaWcaaaa@2E2D@) we can then determine, for each putative target site, the posterior probability that the site is functional given its conservation pattern c→
 MathType@MTEF@5@5@+=feaafiart1ev1aaatCvAUfKttLearuWrP9MDH5MBPbIqV92AaeXatLxBI9gBaebbnrfifHhDYfgasaacH8akY=wiFfYdH8Gipec8Eeeu0xXdbba9frFj0=OqFfea0dXdd9vqai=hGuQ8kuc9pgc9s8qqaq=dirpe0xb9q8qiLsFr0=vr0=vr0dc8meaabaqaciaacaGaaeqabaqabeGadaaakeaacuWGJbWygaWcaaaa@2E0D@. Finally to determine an overall probability that a given 3' UTR is targeted by a given miRNA we combine the posterior probabilities of all sites for the miRNA occurring in the 3' UTR. The reader is again referred to the Methods section for the details of all these procedures.

### Phylogenetic distribution of functional target sites across miRNAs

**Figure 2:**
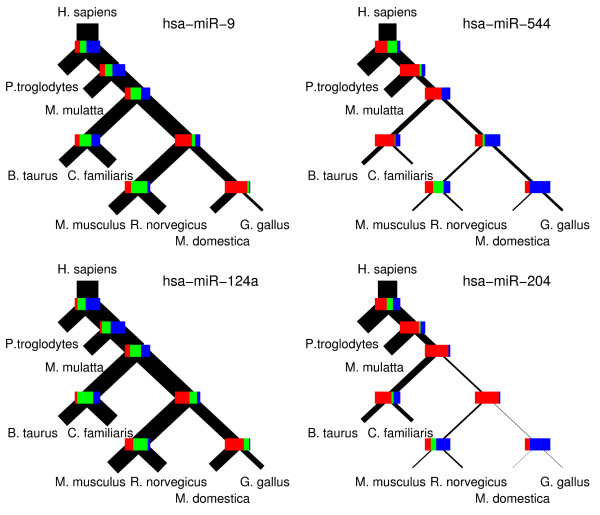
**Examples of inferred phylogenetic distributions of functional target sites**. Comparison of the inferred phylogenetic distribution of functional target sites across vertebrate species (human – H. sapiens, chimp – P. troglodytes, rhesus maccaque – M. mulatta, mouse – M. musculus, rat – R. norvegicus, cow – B. taurus, dog – C. familiaris, opossum – M. domestica, chicken – G. gallus) for 4 different miRNAs. Starting from human at the root the thickness of the branches of the tree represents the fraction of putative target sites inferred to be selected along that branch of the tree. The bars at each internal node indicate what fraction of sites remains under selection in both descending branches (green), only the left descending branch (red), and only the right descending branch (blue). For each of the human miRNAs shown in this figure, there exists at least a miRNA with the same 1–8 "seed" sequence in all vertebrates in the tree.

Note that the estimated distribution over selection patterns *p*(s→
 MathType@MTEF@5@5@+=feaafiart1ev1aaatCvAUfKttLearuWrP9MDH5MBPbIqV92AaeXatLxBI9gBaebbnrfifHhDYfgasaacH8akY=wiFfYdH8Gipec8Eeeu0xXdbba9frFj0=OqFfea0dXdd9vqai=hGuQ8kuc9pgc9s8qqaq=dirpe0xb9q8qiLsFr0=vr0=vr0dc8meaabaqaciaacaGaaeqabaqabeGadaaakeaacuWGZbWCgaWcaaaa@2E2D@) quantifies what fraction of putative sites in the reference species is under selection in each of the possible subsets of other species. That is, *p*(s→
 MathType@MTEF@5@5@+=feaafiart1ev1aaatCvAUfKttLearuWrP9MDH5MBPbIqV92AaeXatLxBI9gBaebbnrfifHhDYfgasaacH8akY=wiFfYdH8Gipec8Eeeu0xXdbba9frFj0=OqFfea0dXdd9vqai=hGuQ8kuc9pgc9s8qqaq=dirpe0xb9q8qiLsFr0=vr0=vr0dc8meaabaqaciaacaGaaeqabaqabeGadaaakeaacuWGZbWCgaWcaaaa@2E2D@) estimates how functional target sites are distributed over the phylogenetic tree. Since we estimate *p*(s→
 MathType@MTEF@5@5@+=feaafiart1ev1aaatCvAUfKttLearuWrP9MDH5MBPbIqV92AaeXatLxBI9gBaebbnrfifHhDYfgasaacH8akY=wiFfYdH8Gipec8Eeeu0xXdbba9frFj0=OqFfea0dXdd9vqai=hGuQ8kuc9pgc9s8qqaq=dirpe0xb9q8qiLsFr0=vr0=vr0dc8meaabaqaciaacaGaaeqabaqabeGadaaakeaacuWGZbWCgaWcaaaa@2E2D@) *independently *for each miRNA, our method allows us to compare how functional sites are distributed across the phylogenetic tree for different miRNAs. In Figure 2 we show the inferred phylogenetic distribution of functional target sites for 4 different human miRNAs. The genes of these miRNAs are conserved across all vertebrates shown in the figure. The precise parameters of the distributions *p*(s→
 MathType@MTEF@5@5@+=feaafiart1ev1aaatCvAUfKttLearuWrP9MDH5MBPbIqV92AaeXatLxBI9gBaebbnrfifHhDYfgasaacH8akY=wiFfYdH8Gipec8Eeeu0xXdbba9frFj0=OqFfea0dXdd9vqai=hGuQ8kuc9pgc9s8qqaq=dirpe0xb9q8qiLsFr0=vr0=vr0dc8meaabaqaciaacaGaaeqabaqabeGadaaakeaacuWGZbWCgaWcaaaa@2E2D@) are represented by the bars at each node with red indicating the fraction of sites that remains under selection in the left descending branch only, blue the fraction that remains under selection in the right descending branch only, and green the fraction that remains under selection in both descending branches. The distribution *p*(s→
 MathType@MTEF@5@5@+=feaafiart1ev1aaatCvAUfKttLearuWrP9MDH5MBPbIqV92AaeXatLxBI9gBaebbnrfifHhDYfgasaacH8akY=wiFfYdH8Gipec8Eeeu0xXdbba9frFj0=OqFfea0dXdd9vqai=hGuQ8kuc9pgc9s8qqaq=dirpe0xb9q8qiLsFr0=vr0=vr0dc8meaabaqaciaacaGaaeqabaqabeGadaaakeaacuWGZbWCgaWcaaaa@2E2D@) is also summarized in the thickness of the branches of each tree. Starting from the root (human) the thickness of each branch indicates what fraction of functional target sites is under selection along that branch. Note that the initial branch leading away from human has the same thickness for all four miRNAs, meaning that the fraction of human target sites that is under selection in at least one of the other species is roughly equal for these 4 miRNAs. However, as the figure shows, the two miRNAs on the left and the two miRNAs on the right differ significantly in the inferred pattern of selection across the tree. In particular, whereas the target sites for the miRNAs on the right (miR-9 and miR-124a) tend to be shared between all mammals, and to some extent with chicken and opossum, the target sites for the miRNAs on the right (miR-544 and miR-205) are shared mostly among primates, but not with other mammals. This suggests that, whereas the target repertoires of miR-9 and miR-124a have been largely conserved since the common ancestor of the mammals, significant changes have occurred in the target repertoires of miR-544 and miR-205 since that time. In Additional file 1 we show the parameters of the inferred selection distributions *p*(s→
 MathType@MTEF@5@5@+=feaafiart1ev1aaatCvAUfKttLearuWrP9MDH5MBPbIqV92AaeXatLxBI9gBaebbnrfifHhDYfgasaacH8akY=wiFfYdH8Gipec8Eeeu0xXdbba9frFj0=OqFfea0dXdd9vqai=hGuQ8kuc9pgc9s8qqaq=dirpe0xb9q8qiLsFr0=vr0=vr0dc8meaabaqaciaacaGaaeqabaqabeGadaaakeaacuWGZbWCgaWcaaaa@2E2D@) for all miRNAs that are conserved across all warm-blooded vertebrates that we considered. These results provide a first comprehensive look into the species-specific targets of miRNAs.

Because we infer different distributions *p*(s→
 MathType@MTEF@5@5@+=feaafiart1ev1aaatCvAUfKttLearuWrP9MDH5MBPbIqV92AaeXatLxBI9gBaebbnrfifHhDYfgasaacH8akY=wiFfYdH8Gipec8Eeeu0xXdbba9frFj0=OqFfea0dXdd9vqai=hGuQ8kuc9pgc9s8qqaq=dirpe0xb9q8qiLsFr0=vr0=vr0dc8meaabaqaciaacaGaaeqabaqabeGadaaakeaacuWGZbWCgaWcaaaa@2E2D@) for different miRNAs, and these distributions enter as priors in the Bayesian procedure, we generally assign different posterior probabilities to sites for different miRNAs, even if these sites have exactly the *same *conservation pattern. For example, in the example above a site for miRNA miR-544 that is only conserved in primates would get considerably higher posterior probability than a site for miR-9 with the same conservation pattern. This is because this conservation pattern corresponds better to the inferred selection pattern of miR-544 than the inferred selection pattern of miR-9.

One of the issues that has been extensively discussed in the miRNA literature is the question of the typical number of functional targets per miRNA, and the related question of what fraction of seed matches in 3' UTRs corresponds to functional target sites. Previous work has indicated that the number of targets per miRNA varies across miRNAs [23]. We believe that the ability of our method to infer species-specific miRNA targeting for each miRNA, allows for a more sensitive and accurate estimation of the total number of functional target sites of each miRNA.

**Figure 3:**
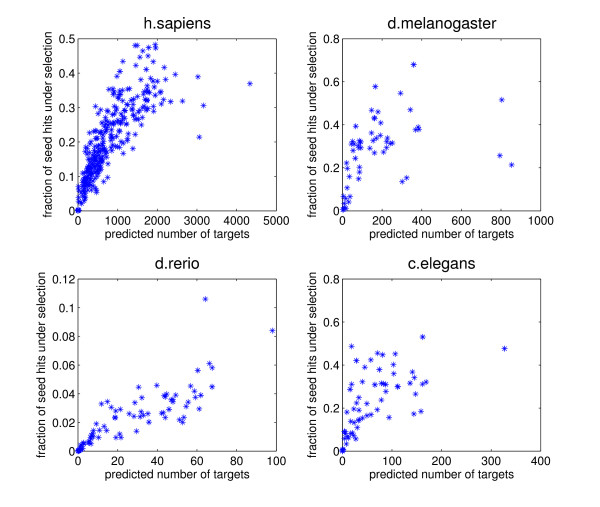
**miRNA seed matches under functional selection**. The fraction *ρ *of seed matches inferred to be under selection (vertical axis) vs. the total number of sites inferred to be under selection in the entire set of mRNAs (horizontal axis) for individual miRNAs. Each star corresponds to one miRNA and each panel corresponds to one clade of species, with the reference species indicated at the top.

There are two independent contributions to the total number of functional target sites for a given miRNA. First, the total number of miRNA seed matches varies from miRNA to miRNA and second, the fraction of seed matches that correspond to functional sites may vary from miRNA to miRNA. The latter can be estimated from the conservation evidence. In particular, the inferred parameter *ρ *of the distribution *p*(s→
 MathType@MTEF@5@5@+=feaafiart1ev1aaatCvAUfKttLearuWrP9MDH5MBPbIqV92AaeXatLxBI9gBaebbnrfifHhDYfgasaacH8akY=wiFfYdH8Gipec8Eeeu0xXdbba9frFj0=OqFfea0dXdd9vqai=hGuQ8kuc9pgc9s8qqaq=dirpe0xb9q8qiLsFr0=vr0=vr0dc8meaabaqaciaacaGaaeqabaqabeGadaaakeaacuWGZbWCgaWcaaaa@2E2D@), corresponds to the fraction of miRNA seed matches that is under selection in the reference species *and *at least one of the other species in the clade. This provides a lower bound on the fraction of seed matches that is functional in the reference species (see Methods). By multiplying this fraction *ρ *by the total number of seed matches for the miRNA we obtain a lower bound on the absolute number of functional target sites for the miRNA. For simplicity we will refer to these as the estimated fraction of functional sites, and the estimated total number of functional sites. Figure 3 shows the estimated fraction of functional sites as a function of the estimated total number of functional target sites, for each clade of species and each miRNA. We infer that the number of functional target sites varies very widely across miRNAs, i.e. from almost zero to several thousands. Similarly, the fraction of target sites under selection varies from close to zero to almost 50% in human, or even more in worms and flies. Overall we find that the average of *ρ *is about 30% for human, fly, and worm, meaning that we predict that at least 30% of miRNA seed matches in these species is functional. The inferred fractions *ρ *are significantly lower in fish. This is most likely because the reference species (Danio rerio) is relatively far (around 120 million years [39,40]) from the other species in the clade (Fugu rubripes and Tetraodon nigroides), so that there is a smaller fraction of sites that is under selection in at least one of the other species. It is intriguing that, for all four clades, there seems to be a correlation between the fraction *ρ *and the inferred total number of functional sites at small values of *ρ*, but no correlation at high values of *ρ*.

The number of predicted sites under selection does not appear to correlate with the breadth of miRNA expression, as among the miRNAs with the largest number of predicted target sites we find some that are highly tissue specific (miR-9 and miR-124 that are expressed in the nervous system [5], and miR-155 that is specific to lymphoid cells [5]) as well some that have broad expression (e.g. the families of miR-29 [9,10,41,42] and miR-30 [41,42]). miR-16, which is ubiquitously expressed [5,41] has an intermediate number of targets (Additional file 2).

### Performance comparison with other methods

To assess the quality of our predictions relative to other methods that have been published to date, we built on the results recently published by Stark et al. [25], who have performed a detailed comparison of the performance of most of the prediction methods that are currently in use on a relatively large set of experimentally tested miRNA-mRNA interactions. This experimental data set has been mostly obtained by the Cohen lab, with a small number of interactions having been tested by other groups. The issues concerning the biases involved with the assembly of this data set have already been discussed by Stark et al. [25], and we will not belabor them here. We will only caution the reader that the accuracy of various different methods on this data set should not be taken as an indication of their accuracy on a random set of miRNA-mRNA interactions. Unfortunately, this unbiased experiment has not been done.

**Figure 4:**
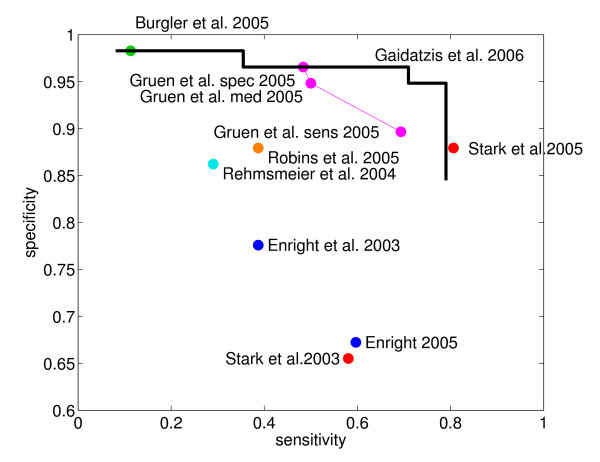
**Performance comparison with other methods**. Comparison of the performance of our method and other published methods on a set of 120 experimentally tested miRNA-mRNA interactions in fly. Specificity (fraction of negatives that are not predicted) is shown as a function of sensitivity (fraction of positives that are predicted) for our method at different cutoffs in posterior probability (black line) and for other methods (colored dots).

Since our method assigns a posterior probability to each predicted site, sets of predictions at different levels of confidence can be obtained by including only sites over a given posterior probability. We created such sets at different thresholds in posterior probability and computed the sensitivity (TPTP+FN
 MathType@MTEF@5@5@+=feaafiart1ev1aaatCvAUfKttLearuWrP9MDH5MBPbIqV92AaeXatLxBI9gBaebbnrfifHhDYfgasaacH8akY=wiFfYdH8Gipec8Eeeu0xXdbba9frFj0=OqFfea0dXdd9vqai=hGuQ8kuc9pgc9s8qqaq=dirpe0xb9q8qiLsFr0=vr0=vr0dc8meaabaqaciaacaGaaeqabaqabeGadaaakeaadaWcaaqaaiabdsfaujabdcfaqbqaaiabdsfaujabdcfaqjabgUcaRiabdAeagjabd6eaobaaaaa@348C@, i.e. the fraction of all true targets that were indeed predicted) and the specificity ((TNFP+TN)
 MathType@MTEF@5@5@+=feaafiart1ev1aaatCvAUfKttLearuWrP9MDH5MBPbIqV92AaeXatLxBI9gBaebbnrfifHhDYfgasaacH8akY=wiFfYdH8Gipec8Eeeu0xXdbba9frFj0=OqFfea0dXdd9vqai=hGuQ8kuc9pgc9s8qqaq=dirpe0xb9q8qiLsFr0=vr0=vr0dc8meaabaqaciaacaGaaeqabaqabeGadaaakeaacqGGOaakdaWcaaqaaiabdsfaujabd6eaobqaaiabdAeagjabdcfaqjabgUcaRiabdsfaujabd6eaobaacqGGPaqkaaa@363A@) i.e. the fraction of all the correct negative predictions) for each set. The results are shown as the black line in Figure 4, which also shows the sensitivities and specificities of other prediction methods [32,36,43-46], as inferred from the published results.

The figure indicates that our method performs as well as the most accurate prediction methods available to date, while maintaining a very high specificity even for high sensitivities. We observe a large overlap between our predicted targets and those predicted by Stark et al. [25] and Grün et al. [23] although there are also substantial numbers of predicted sites that are either specific to our method or specific to one of the other methods. The significant overlap is most likely a reflection of the similarity in the definition of target sites: 7/8-nucleotide seed matches that are conserved across at least some of the other flies account for a large fraction of the predicted sites in all three methods. However, these other methods also consider putative sites with fewer matches in the seed region if they are sufficiently conserved [23] or compensated by matches to the 3' end of the miRNA [25]. The very good accuracy of our predictions indicates that appropriately weighing the evolutionary information enables us to achieve a good performance even with more restrictive definition of putative target sites compared to thee other methods. In particular, we note that from a total of 12,155 high confidence predicted sites (posterior probability *p *≥ 0.5), a substantial proportion, namely 1,953 (16%), are not perfectly conserved in Drosophila pseudoobscura, but are conserved in many of the other flies. Such sites will be missed by methods that only consider strict conservation in D. pseudoobscura.

In Additional file 3 we show a detailed comparison of our predicted target sets for fly miRNAs and those reported by Stark et al. [43] and Grün et al. [23]. We defined a UTR to be a predicted target of a specific miRNA if it had at least 0.5 probability of containing a functional site for the miRNA (see Methods). Because the UTR data sets used by different groups differs to some extent, we have used a conservative scheme of computing the overlap: we have assumed that whenever another method predicted a site in a splice variant of a given gene, all the variants would share the site. Thus, the numbers below represent upper bounds on the extent of overlap between the different methods. Note additionally that the total number of predictions made by other methods may not be the number of predictions reported in the respective studies, but include all the splice variants known to date. The overlap between our predictions and those of Stark et al. [43] and Grün et al. [23] varies significantly between miRNAs. For example, for the bantam miRNAs, which has shown to be involved in the regulation of cell growth [47,48], the overlap is quite large. We predict 140 targets of which 106 (76%) and 121 (86%) occur in the predictions of Stark et al. [25] and Grün et al. [23], respectively. The discrepancy is higher for miR-1, a miRNA required for muscle development [49]. We predict 362 targets of which 252 (70%) and 271 (75%) occur among the predictions of Stark et al. [25] and Grün et al. [23], respectively. Finally, for another microRNA, miR-281, we make only a total of 34 predictions of which only 13 (38%) and 17 (50%) occur among the predictions of Stark et al. [25] and Grün et al. [23], respectively. That is, at least half of our predicted targets are not predicted by the other two algorithms. Unfortunately, the data set of experimentally tested miRNA-mRNA interactions is too small to meaningfully compare the predictions of the different methods for individual miRNAs.

### Location bias of predicted miRNA target sites in UTRs

**Figure 5:**
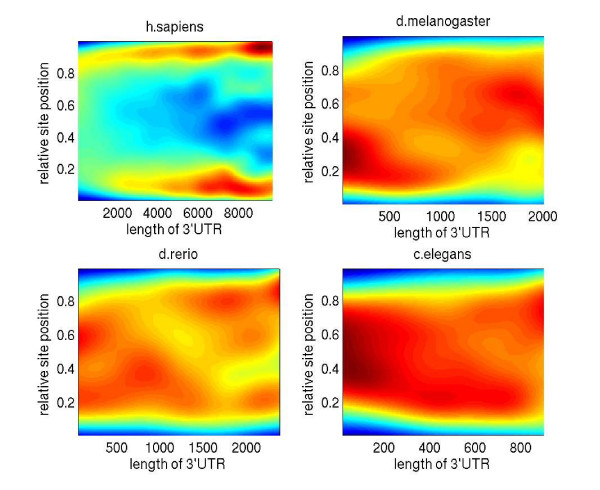
**Location bias of predicted miRNA target sites in UTRs**. Distribution of predicted miRNA target sites in the 3'UTRs. Each predicted miRNA target site is represented by a dot with the x-coordinate corresponding to the length of the associated 3'UTR and the y-coordinate corresponding to the localization of the site within the 3'UTR normalized from 0(start) to 1(end). Gaussian kernels around all the dots were used to create a smooth interpolating density surface. Since the general UTR length distribution is not uniform, we normalized the vertical slices through the 2-D density surface *p*(*x, y*) at each x-coordinate to obtain *p*(*y*|*x*).

**Figure 6:**
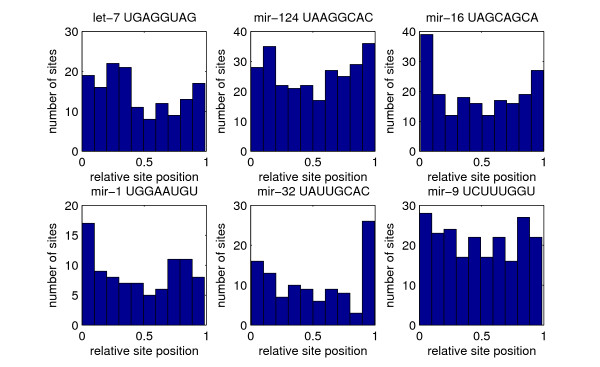
**Location of predicted target sites of individual miRNAs in the 3' UTRs**. Histogram of the relative position (0(start) to 1(end)) of high-probability predicted target sites (posterior probability ≥ 0.5) for 6 individual miRNAs in the human 3'UTRs longer than 4 kb. The identity of the miRNAs and their corresponding seed sequences (positions 1–8 from the 5' end of the mature miRNA) are indicated on each panel.

We next turned to the high-confidence (posterior probability ≥ 0.5) subset of our predicted miRNA target sites and we asked whether we could identify a bias in the location of evolutionarily selected miRNA target sites in the 3' UTRs. Figure 5 shows a heat map representation of the location of these sites along the 3' UTRs in the different clades. In this plot, each predicted miRNA target site is represented as a dot with its x-coordinate being the total length of the 3'UTR in which it resides and its y-coordinate being the relative, normalized position of the site in the UTR. We infer that in all clades, the high-confidence sites tend to avoid the regions immediately after the stop codon as well as the end of the transcript. At the 3' end, this effect could be due to the presence of polyA tails in some of the Refseq transcripts. In human, where the UTRs are much longer than in the other species considered (3,300 of the 22,459 of the human UTRs in our data set were longer than 2 kb), conserved miRNA target sites also tend to avoid the regions in the middle of long UTRs. This pattern is mirrored in the conservation profile across long UTRs, i.e. long UTRs tend to be less conserved in the middle than toward their ends (data not shown). The pattern is also observed at the level of predicted target sites for individual miRNAs (Figure 6), i.e it is not caused by one or two miRNAs with an aberrant target site distribution.

A conceivable explanation for the observed pattern of enrichment of miRNA sites toward the start and end of long UTRs is that a non-negligible proportion of long Refseq UTRs erroneously contains introns. To test this hypothesis we obtained all EST sequences that overlap Refseq UTRs and calculated, for each UTR base, the fraction of all overlapping ESTs in which the base is intronic. As shown in Additional file 4, there is almost no difference between the intron-inclusion profiles for long and short 3' UTRs. That is, the observed enrichment of miRNA sites toward the ends of long 3' UTRs cannot be explained by intron inclusion.

The observed pattern is interesting because it has been argued [25,26] that miRNAs are a major factor driving the evolution of UTR lengths: ubiquitously-expressed genes have short UTRs, while genes whose expression is more restricted and regulated by miRNAs have longer UTRs. Our result suggests a more complicated scenario, in which more strongly conserved miRNA target sites, which have most likely emerged early, are located towards the boundaries of the 3' UTR, the stop codon and the polyadenylation site. This particular location of target sites may influence the likelihood of interaction between the miRNA-containing ribonucleoproteins and other complexes involved in RNA processing and regulation.

### Inference of miRNA function using pathway analysis

To analyze the role that individual miRNAs play in the regulatory networks in human, we have used the KEGG database in which a large fraction of the human genes are assigned to pathways. KEGG provides a mapping between genes and pathways, as well as a reference to the identifier of each of the genes in the Gene database of NCBI. Based on this mapping, as well as on the assignment of Refseq identifiers to Gene identifiers which we obtained from NCBI, we have constructed an assignment between putative miRNA targets and pathways. The resulting dataset consisted of 4, 011 human Refseq transcripts. Using putative target sites with posterior probability of ≥ 0.5, we have determined which pathways are significantly associated with each individual miRNA (see Methods). In particular, for each miRNA/pathway combination we calculated the log-likelihood ratio, given the observed data, of two models: one that assumes that pathway membership and being a predicted target of the miRNA are independent, and one that assumes that these are generally dependent properties.

**Figure 7:**
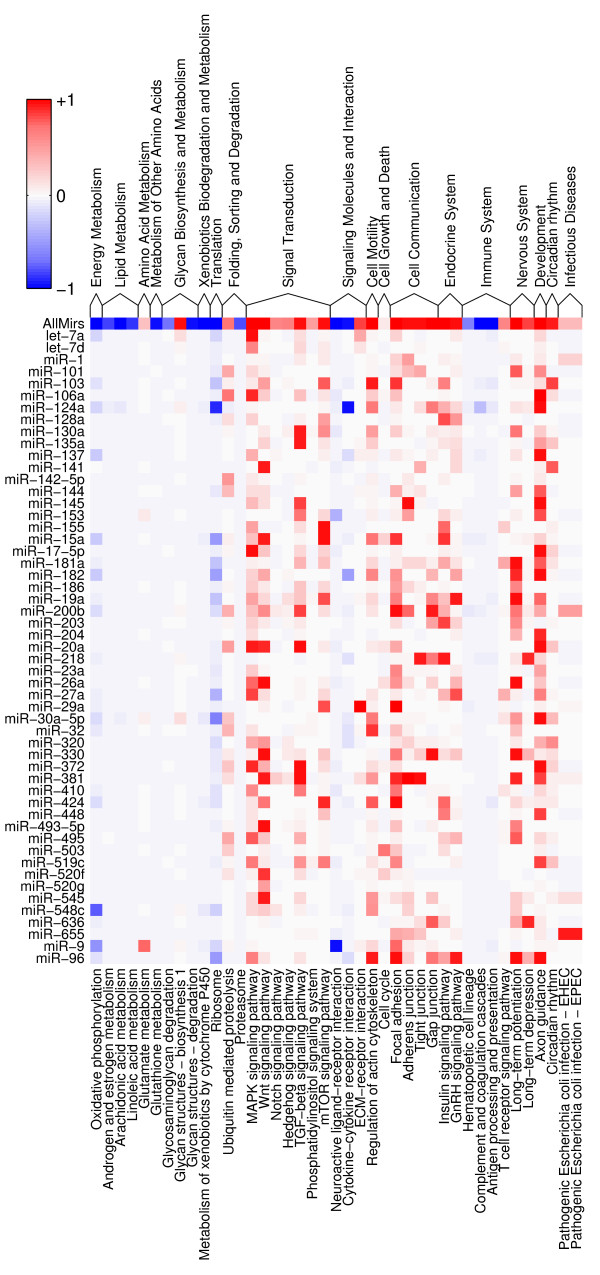
**Pathway analysis**. Representation of individual pathways among the predicted targets of a given miRNA. Each column corresponds to a KEGG pathway and each row to a group of miRNAs with the same seed sequence. Red indicates overrepresentation of the targets of a specific miRNA among the genes in the corresponding pathway, whereas blue indicates depletion. The intensity of the color indicates the posterior probability of the dependent model (see Methods). Pathways have been grouped in larger functional categories according to the KEGG annotation. Only miRNAs with at least one significant association are shown.

Figure 7 shows the results of this analysis for the subset of miRNAs that had at least one significant association (Additional file 5 shows the entire miRNA set). The color scale is centered around a log-likelihood ratio of 0 (white), and the intensity of the color is proportional to the posterior probability of the dependent model. Enrichment of targets in a pathway is shown in red, and depletion of targets in a pathway is shown in blue.

The first thing to note is that, as reported previously [25], genes that are ubiquitous and are involved in basic metabolic functions, tend to be depleted in miRNA target sites. Also noted before is that miRNAs tend to target genes involved in transcription regulation, intercellular communication, cell growth and death and development [22,23,25,30,33,36]. For example, we find that the targets of 19 of the 119 unique miRNA seeds are significantly enriched in the axon guidance pathway. This does not necessarily imply, however, that all these miRNAs are specifically involved in axon guidance. Many of the molecules involved in axon guidance are also involved in delivering spatial cues during the development of other systems, such as for example the cardiovascular system. So it is plausible that, whereas many miRNAs are associated with the axon guidance pathway, different miRNAs may act on different subsets of the mRNAs from this pathway in different tissues. Below we describe some of the most notable associations that we found between specific miRNAs and pathways.

Our method yields the expected associations for miRNAs which are specifically expressed in certain tissues (and presumably regulate processes that are specific to these tissues), or for miRNAs for which targets are already known. miR-124a, whose expression is highly specific to the nervous system, is one of the miRNAs most significantly associated with the axon guidance pathway. Its corresponding targets in this pathway include players with known involvement in nervous system development such as the ephrins B1, B2, and B3, ephrin receptors A2, A3, and B4, semaphorins 5A, 6A, 6C, and 6D, and plexins A3 and B2. As miR-124 is highly expressed in mature neurons, it is possible that its function is to maintain previously established neuronal circuits.

Our results also suggest an involvement of the miR-181 family of miRNAs in nervous system. These miRNAs, whose expression in zebrafish appears to be restricted to the nervous system, thymus and gills [13], have so far been shown to play a role in lymphocyte [50] and muscle [51] development. In our data, they have a set of high confidence targets in the long term potentiation pathway, among which glutamate receptors, calcium/calmodulin-dependent protein kinase II, adenylate cyclate 1, and calmodulin. In fact, calcium/calmodulin kinase II 2 appears to play a role in both memory performance [52] and in activation-induced T cell differentiation [53]. These results may explain the up-to-now puzzling expression pattern of these miRNAs.

The let-7 miRNA, which was recently shown to regulate the let-60 gene in C. elegans and is presumed to regulate the human homologs of let-60, i.e. the Ras genes [35], is most significantly associated with the MAPK pathway, with the NRAS gene and the Ras guanyl releasing protein 1 RasGRP1 being predicted as high confidence targets. Additionally, let-7 is predicted to target several kinases and phosphatases in this pathway, and, importantly for the postulated involvement of let-7 in malignancy, the Fas ligand, TGF*β *receptor I, nerve growth factor and fibroblast growth factor 11.

miR-9 has been described as a brain-specific miRNA [5], and recent evidence suggests that its expression is highest in fetal brain and oligodendrogliomas [54]. The top pathway associated with this miRNA is that of glutamate metabolism, in which miR-9 appears to target glutamate decarboxylase, glutamate dehydrogenase, glutamase, glutamate-cysteine ligase, glutamic-oxaloacetic transaminase 1, as well as glucosamine-phosphate N-acetyltransferase 1, 4-aminobutyrate aminotransferase, and phosphoribosyl pyrophosphate amidotransferase. The second most significant association for miR-9 is with with the focal adhesion pathway, in which many more genes appear to be targeted, among which collagen V *α*1, collagen IV *α*2, integrin 6, tenascin C, talin, trombospondin 2, and vinculin. These targets suggest that miR-9 may be involved in regulating the intercellular communication in the brain and the function of neural circuits. Another group of miRNAs for which we suggest a role a development, in particular in the nervous system, is that of the embryonic miRNAs exemplified by miR-372, initially identified in a study of human embryonic stem cells [10]. These miRNAs appear to be primate-specific. However, the nucleotides at position 2–7, AAGUGC, are shared by the 5' ends of several other miRNAs that are embryonically-expressed and of restricted phylogenetic distribution such as the rodent miR-290 (AAAGUGCC 1–8), miR-291 (AAAGUGCU 1–8), miR-292 (AAAGUGCC 1–8), and miR-294 (AAAGUGCU 1–8), zebrafish miR-430's (U/AAAGUGCU at 1–8), as well as the human miR-302 group (UAAGUGCU at 1–8), miR-373 (GAAGUGCU at 1–8), and most miRNAs of the miR-520 group (AAAGUGCU at 1–8). Of these miRNAs, the study of [55] implicated miR-430 in the nervous system development in zebrafish, although in a subsequent study the authors showed that miR-430 plays a role in the clearance of maternal RNAs [56]. Our results speak to the first proposed role of this class of miRNAs, namely in nervous system development. The miR-372-related miRNAs (AAAGUGCU at 1–8) have a strong predicted association with the axon guidance pathway, where it is predicted to target, among others, the ephrin B2, ephrin receptors A4, A5 and A7, semaphorin 4B, LIM kinase 1, and p21-activated kinase 7. Moreover, at least some of the Smad genes that are part of the top pathway predicted to be targeted by these miRNAs, the TGF*β *pathway, have been implicated in the growth of neurites [57]. Interestingly, the difference A vs. U or G at the first position between the miR-372 and other families mentioned above leads to quite different predictions of targeted pathways. For none of these other miRNAs have we found a pathway that appears to be significantly targeted.

Finally, we were very interested in understanding the function of miR-16 (which shares its seed with the miR-15 group of miRNAs), a miRNA that appears to be ubiquitously expressed at least in mouse [5], and has been implicated in regulation of apoptosis [58] and of mRNA stability [59]. We find that the most significant association of miR-16 is with the mTOR signaling pathway [60], which integrates nutrient-derived signals and controls cell growth. miR-16 appears to target the rapamycin-insensitive companion of mTOR, several ribosomal protein kinases, components of the eukaryotic translation initiation factor 4 (B and E), insulin-like growth factor 1 and others. The second most significant association of this miRNA is with the Wnt pathway, in which it targets several Wnt (Wnt2B, Wnt3A, Wnt5B, Wnt7A), a Wnt inhibitor (WIF1) and cyclin (D1, D2, D3) proteins, and the third most significant association is focal adhesion, where miR-16 appears to target a large number of transcripts that have fundamental role in cell division and cell-cell communication. Some examples are again the cyclins D1, D2, and D3, cell division cycle 42, p21-activated kinase 7 (PAK7), v-akt murine thymoma viral oncogene homolog 3 (AKT3), v-crk sarcoma virus CT10 oncogene homolog (avian)-like (CRKL), mitogen-activated protein kinase kinase 1 (MAP2K1), laminin gamma 1, B-cell CLL/lymphoma 2 (BCL2), and others. These suggest a fundamental role of miR-16 in controlling cell growth and maintaining cell-cell interactions. These functions may explain the observed association between miR-16/miR-15a deletions and chronic lymphocytic leukemia [61], and the slower progression of CLL in mice treated with rapamycin [62].

## Conclusion

As the number of miRNA genes has been growing steadily, especially through high-throughput cloning techniques, the number of experimentally validated targets has been lagging markedly behind. Recently, studies that take advantage of the fact that miRNAs appear to also induce partial degradation of their mRNA targets have used microarray methodology to identify genes whose expression changes upon over-expression or knock-down of individual miRNAs. Typically hundreds of putative targets are identified in such studies but there is only partial overlap between these sets of putative targets and those that are computationally predicted using comparative genomics methods. Computational modeling of miRNA-mRNA interaction and accurate prediction of miRNA target sites therefore remains an important and challenging problem in bioinformatics. In particular, it is still poorly understood what constraints beyond matching of the miRNA seed determine functionality of putative target sites.

In this study, we developed a general method for miRNA target prediction that extends the already available methods in several ways. First, we treat the phylogenetic relationships between species in a rigorous and general way, without any tunable parameters. That is, the Bayesian procedure uniquely determines the posterior probabilities for each conservation pattern and seed type in terms of the observed conservation patterns of target sites for each miRNA. Thus, in contrast to many other target predictions methods which are specifically tailored to operate on a particular clade of species, our method can be applied to any clade of species, and the phylogenetic relations between the species will be automatically taken into account when assessing the significance of the site conservation patterns. This will, for example, enable us to easily update our predictions as more genomes become available, without the need of adapting the method.

Note also that our Bayesian procedure for incorporating information from conservation statistics is generally independent from the "site" definition that we employ and can easily be applied to other target site definitions (see Methods for details). Thus, if a better definition of target sites is developed in the future, for example through a better understanding of the requirements on functional miRNA target sites, then we can easily adapt the method to incorporate conservation statistics in essentially the same way. Most generally put, given a binary function that distinguishes "sites" from "non-sites" in RNA sequences, and given a set of "background frequencies" *p*(c→
 MathType@MTEF@5@5@+=feaafiart1ev1aaatCvAUfKttLearuWrP9MDH5MBPbIqV92AaeXatLxBI9gBaebbnrfifHhDYfgasaacH8akY=wiFfYdH8Gipec8Eeeu0xXdbba9frFj0=OqFfea0dXdd9vqai=hGuQ8kuc9pgc9s8qqaq=dirpe0xb9q8qiLsFr0=vr0=vr0dc8meaabaqaciaacaGaaeqabaqabeGadaaakeaacuWGJbWygaWcaaaa@2E0D@|*bg*) with which sites defined by such a function show conservation pattern c→
 MathType@MTEF@5@5@+=feaafiart1ev1aaatCvAUfKttLearuWrP9MDH5MBPbIqV92AaeXatLxBI9gBaebbnrfifHhDYfgasaacH8akY=wiFfYdH8Gipec8Eeeu0xXdbba9frFj0=OqFfea0dXdd9vqai=hGuQ8kuc9pgc9s8qqaq=dirpe0xb9q8qiLsFr0=vr0=vr0dc8meaabaqaciaacaGaaeqabaqabeGadaaakeaacuWGJbWygaWcaaaa@2E0D@ by chance, we can apply the same methodology to assign posterior probabilities to all putative sites, incorporating the information from the conservation statistics of these sites.

Second, we estimate the evolution of selection pressures on target sites in a miRNA-specific manner. This enables us to correctly treat miRNAs that appeared at different stages in evolution, and whose targets may have undergone different selection pressures in different lineages. In particular, we show that different miRNAs show markedly different distributions of functional target sites across the phylogenetic tree and provide the first comprehensive picture of species-specific and clade-specific miRNA targeting. We have additionally shown that, especially in long 3' UTRs that occur in vertebrates, miRNA target sites show a significant bias toward occurrence near the start and end of the 3' UTR. This suggests the possibility that the choice of a distal polyadenylation site may reduce the activity of a miRNA target cassette in the center of the 3' UTR, while introducing other miRNA target sites close to the new polyA tail.

With respect to the performance of our algorithm, we have shown that in fly, where extensive comparisons of the performance of target prediction algorithms have been done, our method performs at least as well as the most accurate methods available today, with a high specificity over a relatively large range of sensitivities.

Finally, to more robustly infer the function of individual miRNAs, each of whom may target hundreds of transcripts, we developed a method for identifying biochemical pathways that are significantly enriched or depleted in targets of a specific miRNA. We showed that, for well-studied miRNAs, this approach recovers the known functional associations. In addition, this analysis predicts novel pathway associations for a significant number of miRNAs.

## Methods

### Conservation fold enrichment of different seed types

For the data shown in Figure 1 we focused, for each clade, on all miRNAs that occur in all species of the clade. Given that the seed sequence is so important for our inference, we used small RNA cloning data in human to determine the most abundant form of each mature miRNA (Pfeffer et al. [63,64] and M.Zavolan & T.Tuschl, unpublished data), and we used this form in our prediction (Additional file 6). To determine which miRNAs are conserved in the clade we started with miRNA genes annotated in miRBase and searched the genomes of the other species for matches to the mature miRNA. Whenever the mature miRNA mapped with at most one mismatch we consider the mature miRNA conserved in that species. Since our inferences only uses the first 8 nucleotides of a miRNA, we then consider a miRNA seed to be conserved in a species if there exists at least one mature miRNA in that species with the corresponding seed.

For each seed type *t *we located all sites in the 3' UTRs of the reference species that are complementary to a seed of type *t *for any of the conserved miRNAs and then computed the fraction *c*_*t *_of these sites that are conserved in all other species of the clade. We also determined the "background" conservation frequencies *b*_*t *_for each seed type by scanning all 3' UTRs of the reference species and computing the fraction of all sequence segments of the same length as the seed that are conserved in all other species of the clade. Note that all seeds of the same length have the same background frequency *b*_*t*_. This is because we found that this frequency is largely independent of the number of occurrences of a particular sequence segment in the reference species. Finally, the conservation fold enrichment *f*_*t *_of seed type *t *is defined as the ratio of observed and background conservation rates: *f*_*t *_= *c*_*t*_/*b*_*t*_.

### Bayesian phylogenetic miRNA target identification algorithm

For each miRNA and each of the three seed types we identify putative target sites separately and assign a posterior probability to each target site as follows. First we find all "sites" that are complementary to the seed in the 3' UTRs of the reference species. Using pairwise alignments between the reference species and the other species we determine, for each putative site, which other species have the site conserved. An individual site was considered conserved if all the base pairs predicted to form between the miRNA and this site in the reference species could also be formed with the corresponding sites, extracted from the genome alignments, in the other species. This defines a "conservation pattern" for each site, which is a binary vector c→
 MathType@MTEF@5@5@+=feaafiart1ev1aaatCvAUfKttLearuWrP9MDH5MBPbIqV92AaeXatLxBI9gBaebbnrfifHhDYfgasaacH8akY=wiFfYdH8Gipec8Eeeu0xXdbba9frFj0=OqFfea0dXdd9vqai=hGuQ8kuc9pgc9s8qqaq=dirpe0xb9q8qiLsFr0=vr0=vr0dc8meaabaqaciaacaGaaeqabaqabeGadaaakeaacuWGJbWygaWcaaaa@2E0D@ with *c*_*i *_= 1 if the site is conserved in species *i *and *c*_*i *_= 0 if the site is not conserved. For example, for the triplet of worms C. elegans, C. briggsae, and C. remanei, using C. elegans as the reference species, the vector c→
 MathType@MTEF@5@5@+=feaafiart1ev1aaatCvAUfKttLearuWrP9MDH5MBPbIqV92AaeXatLxBI9gBaebbnrfifHhDYfgasaacH8akY=wiFfYdH8Gipec8Eeeu0xXdbba9frFj0=OqFfea0dXdd9vqai=hGuQ8kuc9pgc9s8qqaq=dirpe0xb9q8qiLsFr0=vr0=vr0dc8meaabaqaciaacaGaaeqabaqabeGadaaakeaacuWGJbWygaWcaaaa@2E0D@ = (1, 1) indicates a C. elegans site that is conserved in both other worms, the vector c→
 MathType@MTEF@5@5@+=feaafiart1ev1aaatCvAUfKttLearuWrP9MDH5MBPbIqV92AaeXatLxBI9gBaebbnrfifHhDYfgasaacH8akY=wiFfYdH8Gipec8Eeeu0xXdbba9frFj0=OqFfea0dXdd9vqai=hGuQ8kuc9pgc9s8qqaq=dirpe0xb9q8qiLsFr0=vr0=vr0dc8meaabaqaciaacaGaaeqabaqabeGadaaakeaacuWGJbWygaWcaaaa@2E0D@ = (1, 0) a site conserved only in C. briggsae, the vector c→
 MathType@MTEF@5@5@+=feaafiart1ev1aaatCvAUfKttLearuWrP9MDH5MBPbIqV92AaeXatLxBI9gBaebbnrfifHhDYfgasaacH8akY=wiFfYdH8Gipec8Eeeu0xXdbba9frFj0=OqFfea0dXdd9vqai=hGuQ8kuc9pgc9s8qqaq=dirpe0xb9q8qiLsFr0=vr0=vr0dc8meaabaqaciaacaGaaeqabaqabeGadaaakeaacuWGJbWygaWcaaaa@2E0D@ = (0, 1) a site conserved only in C. remanei, and the vector c→
 MathType@MTEF@5@5@+=feaafiart1ev1aaatCvAUfKttLearuWrP9MDH5MBPbIqV92AaeXatLxBI9gBaebbnrfifHhDYfgasaacH8akY=wiFfYdH8Gipec8Eeeu0xXdbba9frFj0=OqFfea0dXdd9vqai=hGuQ8kuc9pgc9s8qqaq=dirpe0xb9q8qiLsFr0=vr0=vr0dc8meaabaqaciaacaGaaeqabaqabeGadaaakeaacuWGJbWygaWcaaaa@2E0D@ = (0, 0) a site conserved in neither of the other two worms.

The fact that a putative target site is conserved does not necessarily imply that the site is functional. Especially for closely-related species short sequence segments, such as the 7-mers and 8-mers of miRNA seeds, can easily be conserved by chance. This evolutionary dependency between orthologous sites can be taken into account in a number of different ways. For example, in RNAhybrid [32] the *p*-values for orthologous target sites are combined by fitting an "effective" number of orthologous sequences to the observed *p*-value distribution for randomly generated miRNAs. Here we aim to incorporate the conservation statistics in a Bayesian framework that takes the phylogeny of the species explicitly into account and recognizes that a conserved site may be under selection in any of the subsets of species in which the site is conserved. That is, to infer how likely it is that a given putative site with conservation pattern c→
 MathType@MTEF@5@5@+=feaafiart1ev1aaatCvAUfKttLearuWrP9MDH5MBPbIqV92AaeXatLxBI9gBaebbnrfifHhDYfgasaacH8akY=wiFfYdH8Gipec8Eeeu0xXdbba9frFj0=OqFfea0dXdd9vqai=hGuQ8kuc9pgc9s8qqaq=dirpe0xb9q8qiLsFr0=vr0=vr0dc8meaabaqaciaacaGaaeqabaqabeGadaaakeaacuWGJbWygaWcaaaa@2E0D@ is functional, we want to calculate how likely it is to observe this conservation pattern c→
 MathType@MTEF@5@5@+=feaafiart1ev1aaatCvAUfKttLearuWrP9MDH5MBPbIqV92AaeXatLxBI9gBaebbnrfifHhDYfgasaacH8akY=wiFfYdH8Gipec8Eeeu0xXdbba9frFj0=OqFfea0dXdd9vqai=hGuQ8kuc9pgc9s8qqaq=dirpe0xb9q8qiLsFr0=vr0=vr0dc8meaabaqaciaacaGaaeqabaqabeGadaaakeaacuWGJbWygaWcaaaa@2E0D@ given that the site is functional and has been maintained by selection in one or more species, and how likely it is to observe c→
 MathType@MTEF@5@5@+=feaafiart1ev1aaatCvAUfKttLearuWrP9MDH5MBPbIqV92AaeXatLxBI9gBaebbnrfifHhDYfgasaacH8akY=wiFfYdH8Gipec8Eeeu0xXdbba9frFj0=OqFfea0dXdd9vqai=hGuQ8kuc9pgc9s8qqaq=dirpe0xb9q8qiLsFr0=vr0=vr0dc8meaabaqaciaacaGaaeqabaqabeGadaaakeaacuWGJbWygaWcaaaa@2E0D@ in the absence of selection for maintenance of the site.

To this end we first define a "background model" that gives the probabilities *p*(c→
 MathType@MTEF@5@5@+=feaafiart1ev1aaatCvAUfKttLearuWrP9MDH5MBPbIqV92AaeXatLxBI9gBaebbnrfifHhDYfgasaacH8akY=wiFfYdH8Gipec8Eeeu0xXdbba9frFj0=OqFfea0dXdd9vqai=hGuQ8kuc9pgc9s8qqaq=dirpe0xb9q8qiLsFr0=vr0=vr0dc8meaabaqaciaacaGaaeqabaqabeGadaaakeaacuWGJbWygaWcaaaa@2E0D@|*t*, bg) to observe conservation pattern c→
 MathType@MTEF@5@5@+=feaafiart1ev1aaatCvAUfKttLearuWrP9MDH5MBPbIqV92AaeXatLxBI9gBaebbnrfifHhDYfgasaacH8akY=wiFfYdH8Gipec8Eeeu0xXdbba9frFj0=OqFfea0dXdd9vqai=hGuQ8kuc9pgc9s8qqaq=dirpe0xb9q8qiLsFr0=vr0=vr0dc8meaabaqaciaacaGaaeqabaqabeGadaaakeaacuWGJbWygaWcaaaa@2E0D@ "by chance" for a seed of type *t*, i.e. a particular 7-mer or 8-mer. By "conservation by chance" we mean that there is no specific selection for maintaining the complementarity of the region in question to the 5' end of the miRNA. We did not, however, use a background model that simply reflects the probabilities to observe different conservation patterns under neutral evolution. Any particular putative target site may overlap or be part of a site that is functional for some other reason, and may therefore be more conserved than would be expected under neutral evolution alone. Therefore, to estimate the background probabilities *p*(c→
 MathType@MTEF@5@5@+=feaafiart1ev1aaatCvAUfKttLearuWrP9MDH5MBPbIqV92AaeXatLxBI9gBaebbnrfifHhDYfgasaacH8akY=wiFfYdH8Gipec8Eeeu0xXdbba9frFj0=OqFfea0dXdd9vqai=hGuQ8kuc9pgc9s8qqaq=dirpe0xb9q8qiLsFr0=vr0=vr0dc8meaabaqaciaacaGaaeqabaqabeGadaaakeaacuWGJbWygaWcaaaa@2E0D@|*t*, bg) we calculated the overall frequencies with which all conservation patterns c→
 MathType@MTEF@5@5@+=feaafiart1ev1aaatCvAUfKttLearuWrP9MDH5MBPbIqV92AaeXatLxBI9gBaebbnrfifHhDYfgasaacH8akY=wiFfYdH8Gipec8Eeeu0xXdbba9frFj0=OqFfea0dXdd9vqai=hGuQ8kuc9pgc9s8qqaq=dirpe0xb9q8qiLsFr0=vr0=vr0dc8meaabaqaciaacaGaaeqabaqabeGadaaakeaacuWGJbWygaWcaaaa@2E0D@ occur in the alignments, averaged over all 8-mers for the 1–8 seed type, and averaged over all 7-mers for the 1–7 and 2–8 seed types. In previous work others [22] have estimated background frequencies of conserved seed matches independently for seeds that have different absolute frequencies in the 3' UTRs of the reference species. We, in contrast, only require the *relative *frequencies of different conservation patterns, and we have observed that these are largely independent of the absolute frequency of the seed match. Note that for a clade consisting of the reference species and *g *other species, we are estimating the relative frequencies of 2^*g *^possible conservation patterns for each seed type. Further subdividing these 2^*g *^different conservation patterns by the absolute frequency of the seed match would reduce the amount of data available per seed too much for an accurate estimation of all the parameters.

We next calculated how likely it is to observe different conservation patterns c→
 MathType@MTEF@5@5@+=feaafiart1ev1aaatCvAUfKttLearuWrP9MDH5MBPbIqV92AaeXatLxBI9gBaebbnrfifHhDYfgasaacH8akY=wiFfYdH8Gipec8Eeeu0xXdbba9frFj0=OqFfea0dXdd9vqai=hGuQ8kuc9pgc9s8qqaq=dirpe0xb9q8qiLsFr0=vr0=vr0dc8meaabaqaciaacaGaaeqabaqabeGadaaakeaacuWGJbWygaWcaaaa@2E0D@ given that the putative target site is functional in at least one of the species. To this end we had to quantify the effect of selection on functional target sites. This is very difficult to do in complete generality. For example, one would generally expect that mutations that destroy functional target sites can have wildly varying effects on fitness with some sites being almost lethal when destroyed and others having only very mild deleterious effects. In addition these fitness effects will generally differ from species to the species, even for orthologous functional target sites. Of course target sites can also be spontaneously created through mutations in 3' UTRs, and in some cases these will act as functional target sites that can have either beneficial or deleterious effects. Thus, the rates at which orthologous target sites appear and disappear through evolution is a complex function of fluctuating selection pressures of which we know virtually nothing. In order to be able to calculate meaningful probabilities for observing different conservation patterns c→
 MathType@MTEF@5@5@+=feaafiart1ev1aaatCvAUfKttLearuWrP9MDH5MBPbIqV92AaeXatLxBI9gBaebbnrfifHhDYfgasaacH8akY=wiFfYdH8Gipec8Eeeu0xXdbba9frFj0=OqFfea0dXdd9vqai=hGuQ8kuc9pgc9s8qqaq=dirpe0xb9q8qiLsFr0=vr0=vr0dc8meaabaqaciaacaGaaeqabaqabeGadaaakeaacuWGJbWygaWcaaaa@2E0D@ for functional sites we therefore make the following simplifying assumptions. First, we assume that given a set of conserved putative target sites, each of the conserved sites can be either "functional" or "nonfunctional". In this context "functional" means that selection has acted to ensure that the target site remains conserved and "nonfunctional" means that the target site has evolved according to the background model. To take the worm example, if a functional C. elegans site is functional in both other worms as well, than the site will necessarily be conserved in both, i.e. we will have c→
 MathType@MTEF@5@5@+=feaafiart1ev1aaatCvAUfKttLearuWrP9MDH5MBPbIqV92AaeXatLxBI9gBaebbnrfifHhDYfgasaacH8akY=wiFfYdH8Gipec8Eeeu0xXdbba9frFj0=OqFfea0dXdd9vqai=hGuQ8kuc9pgc9s8qqaq=dirpe0xb9q8qiLsFr0=vr0=vr0dc8meaabaqaciaacaGaaeqabaqabeGadaaakeaacuWGJbWygaWcaaaa@2E0D@ = (1, 1). If the site is functional in C. briggsae only, then we might observe either c→
 MathType@MTEF@5@5@+=feaafiart1ev1aaatCvAUfKttLearuWrP9MDH5MBPbIqV92AaeXatLxBI9gBaebbnrfifHhDYfgasaacH8akY=wiFfYdH8Gipec8Eeeu0xXdbba9frFj0=OqFfea0dXdd9vqai=hGuQ8kuc9pgc9s8qqaq=dirpe0xb9q8qiLsFr0=vr0=vr0dc8meaabaqaciaacaGaaeqabaqabeGadaaakeaacuWGJbWygaWcaaaa@2E0D@ = (1, 0) or c→
 MathType@MTEF@5@5@+=feaafiart1ev1aaatCvAUfKttLearuWrP9MDH5MBPbIqV92AaeXatLxBI9gBaebbnrfifHhDYfgasaacH8akY=wiFfYdH8Gipec8Eeeu0xXdbba9frFj0=OqFfea0dXdd9vqai=hGuQ8kuc9pgc9s8qqaq=dirpe0xb9q8qiLsFr0=vr0=vr0dc8meaabaqaciaacaGaaeqabaqabeGadaaakeaacuWGJbWygaWcaaaa@2E0D@ = (1, 1), because the site is necessarily conserved in C. briggsae, and it may still remain unmutated by chance in C. remanei.

Thus, in general we consider all possible "selection patterns" for the site across the different species. Like the conservation pattern, a selection pattern s→
 MathType@MTEF@5@5@+=feaafiart1ev1aaatCvAUfKttLearuWrP9MDH5MBPbIqV92AaeXatLxBI9gBaebbnrfifHhDYfgasaacH8akY=wiFfYdH8Gipec8Eeeu0xXdbba9frFj0=OqFfea0dXdd9vqai=hGuQ8kuc9pgc9s8qqaq=dirpe0xb9q8qiLsFr0=vr0=vr0dc8meaabaqaciaacaGaaeqabaqabeGadaaakeaacuWGZbWCgaWcaaaa@2E2D@ is a binary vector with *s*_*i *_= 1 if the site is functional (under selection) in species *i*, and *s*_*i *_= 0 otherwise. We calculate the probabilities *p*(c→
 MathType@MTEF@5@5@+=feaafiart1ev1aaatCvAUfKttLearuWrP9MDH5MBPbIqV92AaeXatLxBI9gBaebbnrfifHhDYfgasaacH8akY=wiFfYdH8Gipec8Eeeu0xXdbba9frFj0=OqFfea0dXdd9vqai=hGuQ8kuc9pgc9s8qqaq=dirpe0xb9q8qiLsFr0=vr0=vr0dc8meaabaqaciaacaGaaeqabaqabeGadaaakeaacuWGJbWygaWcaaaa@2E0D@|*t*, s→
 MathType@MTEF@5@5@+=feaafiart1ev1aaatCvAUfKttLearuWrP9MDH5MBPbIqV92AaeXatLxBI9gBaebbnrfifHhDYfgasaacH8akY=wiFfYdH8Gipec8Eeeu0xXdbba9frFj0=OqFfea0dXdd9vqai=hGuQ8kuc9pgc9s8qqaq=dirpe0xb9q8qiLsFr0=vr0=vr0dc8meaabaqaciaacaGaaeqabaqabeGadaaakeaacuWGZbWCgaWcaaaa@2E2D@) to observe conservation pattern c→
 MathType@MTEF@5@5@+=feaafiart1ev1aaatCvAUfKttLearuWrP9MDH5MBPbIqV92AaeXatLxBI9gBaebbnrfifHhDYfgasaacH8akY=wiFfYdH8Gipec8Eeeu0xXdbba9frFj0=OqFfea0dXdd9vqai=hGuQ8kuc9pgc9s8qqaq=dirpe0xb9q8qiLsFr0=vr0=vr0dc8meaabaqaciaacaGaaeqabaqabeGadaaakeaacuWGJbWygaWcaaaa@2E0D@ given selection pattern s→
 MathType@MTEF@5@5@+=feaafiart1ev1aaatCvAUfKttLearuWrP9MDH5MBPbIqV92AaeXatLxBI9gBaebbnrfifHhDYfgasaacH8akY=wiFfYdH8Gipec8Eeeu0xXdbba9frFj0=OqFfea0dXdd9vqai=hGuQ8kuc9pgc9s8qqaq=dirpe0xb9q8qiLsFr0=vr0=vr0dc8meaabaqaciaacaGaaeqabaqabeGadaaakeaacuWGZbWCgaWcaaaa@2E2D@ (and seed type *t*) as follows. Let *C*(s→
 MathType@MTEF@5@5@+=feaafiart1ev1aaatCvAUfKttLearuWrP9MDH5MBPbIqV92AaeXatLxBI9gBaebbnrfifHhDYfgasaacH8akY=wiFfYdH8Gipec8Eeeu0xXdbba9frFj0=OqFfea0dXdd9vqai=hGuQ8kuc9pgc9s8qqaq=dirpe0xb9q8qiLsFr0=vr0=vr0dc8meaabaqaciaacaGaaeqabaqabeGadaaakeaacuWGZbWCgaWcaaaa@2E2D@) denote the set of all conservation patterns c→
 MathType@MTEF@5@5@+=feaafiart1ev1aaatCvAUfKttLearuWrP9MDH5MBPbIqV92AaeXatLxBI9gBaebbnrfifHhDYfgasaacH8akY=wiFfYdH8Gipec8Eeeu0xXdbba9frFj0=OqFfea0dXdd9vqai=hGuQ8kuc9pgc9s8qqaq=dirpe0xb9q8qiLsFr0=vr0=vr0dc8meaabaqaciaacaGaaeqabaqabeGadaaakeaacuWGJbWygaWcaaaa@2E0D@ that are consistent with the selection pattern s→
 MathType@MTEF@5@5@+=feaafiart1ev1aaatCvAUfKttLearuWrP9MDH5MBPbIqV92AaeXatLxBI9gBaebbnrfifHhDYfgasaacH8akY=wiFfYdH8Gipec8Eeeu0xXdbba9frFj0=OqFfea0dXdd9vqai=hGuQ8kuc9pgc9s8qqaq=dirpe0xb9q8qiLsFr0=vr0=vr0dc8meaabaqaciaacaGaaeqabaqabeGadaaakeaacuWGZbWCgaWcaaaa@2E2D@. To be consistent with the selection pattern, the site needs to be conserved in all species in which it is presumed to be under selection, i.e. for all c→
 MathType@MTEF@5@5@+=feaafiart1ev1aaatCvAUfKttLearuWrP9MDH5MBPbIqV92AaeXatLxBI9gBaebbnrfifHhDYfgasaacH8akY=wiFfYdH8Gipec8Eeeu0xXdbba9frFj0=OqFfea0dXdd9vqai=hGuQ8kuc9pgc9s8qqaq=dirpe0xb9q8qiLsFr0=vr0=vr0dc8meaabaqaciaacaGaaeqabaqabeGadaaakeaacuWGJbWygaWcaaaa@2E0D@ in *C*(s→
 MathType@MTEF@5@5@+=feaafiart1ev1aaatCvAUfKttLearuWrP9MDH5MBPbIqV92AaeXatLxBI9gBaebbnrfifHhDYfgasaacH8akY=wiFfYdH8Gipec8Eeeu0xXdbba9frFj0=OqFfea0dXdd9vqai=hGuQ8kuc9pgc9s8qqaq=dirpe0xb9q8qiLsFr0=vr0=vr0dc8meaabaqaciaacaGaaeqabaqabeGadaaakeaacuWGZbWCgaWcaaaa@2E2D@) we have that *c*_*i *_= 1 for all *i *for which *s*_*i *_= 1. The probability *p*(c→
 MathType@MTEF@5@5@+=feaafiart1ev1aaatCvAUfKttLearuWrP9MDH5MBPbIqV92AaeXatLxBI9gBaebbnrfifHhDYfgasaacH8akY=wiFfYdH8Gipec8Eeeu0xXdbba9frFj0=OqFfea0dXdd9vqai=hGuQ8kuc9pgc9s8qqaq=dirpe0xb9q8qiLsFr0=vr0=vr0dc8meaabaqaciaacaGaaeqabaqabeGadaaakeaacuWGJbWygaWcaaaa@2E0D@|*t*, s→
 MathType@MTEF@5@5@+=feaafiart1ev1aaatCvAUfKttLearuWrP9MDH5MBPbIqV92AaeXatLxBI9gBaebbnrfifHhDYfgasaacH8akY=wiFfYdH8Gipec8Eeeu0xXdbba9frFj0=OqFfea0dXdd9vqai=hGuQ8kuc9pgc9s8qqaq=dirpe0xb9q8qiLsFr0=vr0=vr0dc8meaabaqaciaacaGaaeqabaqabeGadaaakeaacuWGZbWCgaWcaaaa@2E2D@) is then given by

p(c→|t,s→)=p(c→|t,bg)∑c→′∈C(s→)p(c→′|t,bg).     (1)
 MathType@MTEF@5@5@+=feaafiart1ev1aaatCvAUfKttLearuWrP9MDH5MBPbIqV92AaeXatLxBI9gBaebbnrfifHhDYfgasaacH8akY=wiFfYdH8Gipec8Eeeu0xXdbba9frFj0=OqFfea0dXdd9vqai=hGuQ8kuc9pgc9s8qqaq=dirpe0xb9q8qiLsFr0=vr0=vr0dc8meaabaqaciaacaGaaeqabaqabeGadaaakeaacqWGWbaCcqGGOaakcuWGJbWygaWcaiabcYha8jabdsha0jabcYcaSiqbdohaZzaalaGaeiykaKIaeyypa0ZaaSaaaeaacqWGWbaCcqGGOaakcuWGJbWygaWcaiabcYha8jabdsha0jabcYcaSiabbkgaIjabbEgaNjabcMcaPaqaamaaqababaGaemiCaaNaeiikaGIafm4yamMbaSGbauaacqGG8baFcqWG0baDcqGGSaalcqqGIbGycqqGNbWzcqGGPaqkaSqaaiqbdogaJzaalyaafaGaeyicI4Saem4qamKaeiikaGIafm4CamNbaSaacqGGPaqkaeqaniabggHiLdaaaOGaeiOla4IaaCzcaiaaxMaadaqadaqaaiabigdaXaGaayjkaiaawMcaaaaa@5B35@

Note that *p*(c→
 MathType@MTEF@5@5@+=feaafiart1ev1aaatCvAUfKttLearuWrP9MDH5MBPbIqV92AaeXatLxBI9gBaebbnrfifHhDYfgasaacH8akY=wiFfYdH8Gipec8Eeeu0xXdbba9frFj0=OqFfea0dXdd9vqai=hGuQ8kuc9pgc9s8qqaq=dirpe0xb9q8qiLsFr0=vr0=vr0dc8meaabaqaciaacaGaaeqabaqabeGadaaakeaacuWGJbWygaWcaaaa@2E0D@|*t*, s→
 MathType@MTEF@5@5@+=feaafiart1ev1aaatCvAUfKttLearuWrP9MDH5MBPbIqV92AaeXatLxBI9gBaebbnrfifHhDYfgasaacH8akY=wiFfYdH8Gipec8Eeeu0xXdbba9frFj0=OqFfea0dXdd9vqai=hGuQ8kuc9pgc9s8qqaq=dirpe0xb9q8qiLsFr0=vr0=vr0dc8meaabaqaciaacaGaaeqabaqabeGadaaakeaacuWGZbWCgaWcaaaa@2E2D@) is just the probability that the site is conserved by chance in those species which have *c*_*i *_= 1 but are not under selection, i.e. *s*_*i *_= 0.

Finally, we need to quantify how likely it is *a priori *that a target site in the reference species will be under selection in a particular subset of the other species. That is, we need a prior probability distribution *p*(s→
 MathType@MTEF@5@5@+=feaafiart1ev1aaatCvAUfKttLearuWrP9MDH5MBPbIqV92AaeXatLxBI9gBaebbnrfifHhDYfgasaacH8akY=wiFfYdH8Gipec8Eeeu0xXdbba9frFj0=OqFfea0dXdd9vqai=hGuQ8kuc9pgc9s8qqaq=dirpe0xb9q8qiLsFr0=vr0=vr0dc8meaabaqaciaacaGaaeqabaqabeGadaaakeaacuWGZbWCgaWcaaaa@2E2D@) that gives the probability that a miRNA site will be under selection in all species *i *for which *s*_*i *_= 1. One of the key novel features of our model is that we allow this prior distribution *p*(s→
 MathType@MTEF@5@5@+=feaafiart1ev1aaatCvAUfKttLearuWrP9MDH5MBPbIqV92AaeXatLxBI9gBaebbnrfifHhDYfgasaacH8akY=wiFfYdH8Gipec8Eeeu0xXdbba9frFj0=OqFfea0dXdd9vqai=hGuQ8kuc9pgc9s8qqaq=dirpe0xb9q8qiLsFr0=vr0=vr0dc8meaabaqaciaacaGaaeqabaqabeGadaaakeaacuWGZbWCgaWcaaaa@2E2D@) to vary between different miRNAs. We thus take into account the species- or clade-specific conservation of functional targets, i.e. that the reference species may share functional target sites with different subsets of species for different miRNAs.

For each miRNA we need to estimate the prior probabilities *p*(s→
 MathType@MTEF@5@5@+=feaafiart1ev1aaatCvAUfKttLearuWrP9MDH5MBPbIqV92AaeXatLxBI9gBaebbnrfifHhDYfgasaacH8akY=wiFfYdH8Gipec8Eeeu0xXdbba9frFj0=OqFfea0dXdd9vqai=hGuQ8kuc9pgc9s8qqaq=dirpe0xb9q8qiLsFr0=vr0=vr0dc8meaabaqaciaacaGaaeqabaqabeGadaaakeaacuWGZbWCgaWcaaaa@2E2D@) of all possible selection patterns. That is, we need to estimate what fraction of putative sites in the reference species is under selection in each possible subset s→
 MathType@MTEF@5@5@+=feaafiart1ev1aaatCvAUfKttLearuWrP9MDH5MBPbIqV92AaeXatLxBI9gBaebbnrfifHhDYfgasaacH8akY=wiFfYdH8Gipec8Eeeu0xXdbba9frFj0=OqFfea0dXdd9vqai=hGuQ8kuc9pgc9s8qqaq=dirpe0xb9q8qiLsFr0=vr0=vr0dc8meaabaqaciaacaGaaeqabaqabeGadaaakeaacuWGZbWCgaWcaaaa@2E2D@ of the other species. To do this we can first use the conservation of the miRNA *gene*. That is, if the miRNA *gene *is not conserved in a given species *i*, then we will assume that sites for this miRNA cannot possibly be under selection in species *i*. Thus, for every miRNA in the reference species we check which of the other species contains a miRNA with the same seed. When then set *p*(s→
 MathType@MTEF@5@5@+=feaafiart1ev1aaatCvAUfKttLearuWrP9MDH5MBPbIqV92AaeXatLxBI9gBaebbnrfifHhDYfgasaacH8akY=wiFfYdH8Gipec8Eeeu0xXdbba9frFj0=OqFfea0dXdd9vqai=hGuQ8kuc9pgc9s8qqaq=dirpe0xb9q8qiLsFr0=vr0=vr0dc8meaabaqaciaacaGaaeqabaqabeGadaaakeaacuWGZbWCgaWcaaaa@2E2D@) = 0 for all vectors s→
 MathType@MTEF@5@5@+=feaafiart1ev1aaatCvAUfKttLearuWrP9MDH5MBPbIqV92AaeXatLxBI9gBaebbnrfifHhDYfgasaacH8akY=wiFfYdH8Gipec8Eeeu0xXdbba9frFj0=OqFfea0dXdd9vqai=hGuQ8kuc9pgc9s8qqaq=dirpe0xb9q8qiLsFr0=vr0=vr0dc8meaabaqaciaacaGaaeqabaqabeGadaaakeaacuWGZbWCgaWcaaaa@2E2D@ in which the site is presumed under selection in a species that does not contain the miRNA. Note that, although unlikely, it is in principle conceivable that problems with the genome assembly of one of the species causes us to miss the ortholog of a particular miRNA gene. This will result in the conservation information from this species to be ignored for this particular miRNA.

The most general approach to estimating *p*(s→
 MathType@MTEF@5@5@+=feaafiart1ev1aaatCvAUfKttLearuWrP9MDH5MBPbIqV92AaeXatLxBI9gBaebbnrfifHhDYfgasaacH8akY=wiFfYdH8Gipec8Eeeu0xXdbba9frFj0=OqFfea0dXdd9vqai=hGuQ8kuc9pgc9s8qqaq=dirpe0xb9q8qiLsFr0=vr0=vr0dc8meaabaqaciaacaGaaeqabaqabeGadaaakeaacuWGZbWCgaWcaaaa@2E2D@) would now be to simply find the distribution *p*(s→
 MathType@MTEF@5@5@+=feaafiart1ev1aaatCvAUfKttLearuWrP9MDH5MBPbIqV92AaeXatLxBI9gBaebbnrfifHhDYfgasaacH8akY=wiFfYdH8Gipec8Eeeu0xXdbba9frFj0=OqFfea0dXdd9vqai=hGuQ8kuc9pgc9s8qqaq=dirpe0xb9q8qiLsFr0=vr0=vr0dc8meaabaqaciaacaGaaeqabaqabeGadaaakeaacuWGZbWCgaWcaaaa@2E2D@) that has overall maximum likelihood given the data. Formally, the probability *p*(c→
 MathType@MTEF@5@5@+=feaafiart1ev1aaatCvAUfKttLearuWrP9MDH5MBPbIqV92AaeXatLxBI9gBaebbnrfifHhDYfgasaacH8akY=wiFfYdH8Gipec8Eeeu0xXdbba9frFj0=OqFfea0dXdd9vqai=hGuQ8kuc9pgc9s8qqaq=dirpe0xb9q8qiLsFr0=vr0=vr0dc8meaabaqaciaacaGaaeqabaqabeGadaaakeaacuWGJbWygaWcaaaa@2E0D@, *t*) to observe the conservation pattern c→
 MathType@MTEF@5@5@+=feaafiart1ev1aaatCvAUfKttLearuWrP9MDH5MBPbIqV92AaeXatLxBI9gBaebbnrfifHhDYfgasaacH8akY=wiFfYdH8Gipec8Eeeu0xXdbba9frFj0=OqFfea0dXdd9vqai=hGuQ8kuc9pgc9s8qqaq=dirpe0xb9q8qiLsFr0=vr0=vr0dc8meaabaqaciaacaGaaeqabaqabeGadaaakeaacuWGJbWygaWcaaaa@2E0D@ for a given putative target site of seed type *t *is given by summing over all possible selection patterns s→
 MathType@MTEF@5@5@+=feaafiart1ev1aaatCvAUfKttLearuWrP9MDH5MBPbIqV92AaeXatLxBI9gBaebbnrfifHhDYfgasaacH8akY=wiFfYdH8Gipec8Eeeu0xXdbba9frFj0=OqFfea0dXdd9vqai=hGuQ8kuc9pgc9s8qqaq=dirpe0xb9q8qiLsFr0=vr0=vr0dc8meaabaqaciaacaGaaeqabaqabeGadaaakeaacuWGZbWCgaWcaaaa@2E2D@:

p(c→,t)=∑s→∈Sp(c→|t,s→)p(s→),     (2)
 MathType@MTEF@5@5@+=feaafiart1ev1aaatCvAUfKttLearuWrP9MDH5MBPbIqV92AaeXatLxBI9gBaebbnrfifHhDYfgasaacH8akY=wiFfYdH8Gipec8Eeeu0xXdbba9frFj0=OqFfea0dXdd9vqai=hGuQ8kuc9pgc9s8qqaq=dirpe0xb9q8qiLsFr0=vr0=vr0dc8meaabaqaciaacaGaaeqabaqabeGadaaakeaacqWGWbaCcqGGOaakcuWGJbWygaWcaiabcYcaSiabdsha0jabcMcaPiabg2da9maaqafabaGaemiCaaNaeiikaGIafm4yamMbaSaacqGG8baFcqWG0baDcqGGSaalcuWGZbWCgaWcaiabcMcaPiabdchaWjabcIcaOiqbdohaZzaalaGaeiykaKcaleaacuWGZbWCgaWcaiabgIGiolabdofatbqab0GaeyyeIuoakiabcYcaSiaaxMaacaWLjaWaaeWaaeaacqaIYaGmaiaawIcacaGLPaaaaaa@4DE9@

where *S *is the set of all selection patterns that are consistent with the miRNA gene conservation pattern, *p*(c→
 MathType@MTEF@5@5@+=feaafiart1ev1aaatCvAUfKttLearuWrP9MDH5MBPbIqV92AaeXatLxBI9gBaebbnrfifHhDYfgasaacH8akY=wiFfYdH8Gipec8Eeeu0xXdbba9frFj0=OqFfea0dXdd9vqai=hGuQ8kuc9pgc9s8qqaq=dirpe0xb9q8qiLsFr0=vr0=vr0dc8meaabaqaciaacaGaaeqabaqabeGadaaakeaacuWGJbWygaWcaaaa@2E0D@|*t*, s→
 MathType@MTEF@5@5@+=feaafiart1ev1aaatCvAUfKttLearuWrP9MDH5MBPbIqV92AaeXatLxBI9gBaebbnrfifHhDYfgasaacH8akY=wiFfYdH8Gipec8Eeeu0xXdbba9frFj0=OqFfea0dXdd9vqai=hGuQ8kuc9pgc9s8qqaq=dirpe0xb9q8qiLsFr0=vr0=vr0dc8meaabaqaciaacaGaaeqabaqabeGadaaakeaacuWGZbWCgaWcaaaa@2E2D@) is given by equation (1), and *p*(s→
 MathType@MTEF@5@5@+=feaafiart1ev1aaatCvAUfKttLearuWrP9MDH5MBPbIqV92AaeXatLxBI9gBaebbnrfifHhDYfgasaacH8akY=wiFfYdH8Gipec8Eeeu0xXdbba9frFj0=OqFfea0dXdd9vqai=hGuQ8kuc9pgc9s8qqaq=dirpe0xb9q8qiLsFr0=vr0=vr0dc8meaabaqaciaacaGaaeqabaqabeGadaaakeaacuWGZbWCgaWcaaaa@2E2D@) is the prior probability distribution over selection patterns which we want to estimate. Let *n*(c→
 MathType@MTEF@5@5@+=feaafiart1ev1aaatCvAUfKttLearuWrP9MDH5MBPbIqV92AaeXatLxBI9gBaebbnrfifHhDYfgasaacH8akY=wiFfYdH8Gipec8Eeeu0xXdbba9frFj0=OqFfea0dXdd9vqai=hGuQ8kuc9pgc9s8qqaq=dirpe0xb9q8qiLsFr0=vr0=vr0dc8meaabaqaciaacaGaaeqabaqabeGadaaakeaacuWGJbWygaWcaaaa@2E0D@, *t*) denote the number of occurrences of putative target sites of seed type *t *that have conservation pattern c→
 MathType@MTEF@5@5@+=feaafiart1ev1aaatCvAUfKttLearuWrP9MDH5MBPbIqV92AaeXatLxBI9gBaebbnrfifHhDYfgasaacH8akY=wiFfYdH8Gipec8Eeeu0xXdbba9frFj0=OqFfea0dXdd9vqai=hGuQ8kuc9pgc9s8qqaq=dirpe0xb9q8qiLsFr0=vr0=vr0dc8meaabaqaciaacaGaaeqabaqabeGadaaakeaacuWGJbWygaWcaaaa@2E0D@. The likelihood *L *given the data, i.e. the observed counts *n*(c→
 MathType@MTEF@5@5@+=feaafiart1ev1aaatCvAUfKttLearuWrP9MDH5MBPbIqV92AaeXatLxBI9gBaebbnrfifHhDYfgasaacH8akY=wiFfYdH8Gipec8Eeeu0xXdbba9frFj0=OqFfea0dXdd9vqai=hGuQ8kuc9pgc9s8qqaq=dirpe0xb9q8qiLsFr0=vr0=vr0dc8meaabaqaciaacaGaaeqabaqabeGadaaakeaacuWGJbWygaWcaaaa@2E0D@, *t*), is then given by

L=∏c→,tp(c→,t)n(c→,t).     (3)
 MathType@MTEF@5@5@+=feaafiart1ev1aaatCvAUfKttLearuWrP9MDH5MBPbIqV92AaeXatLxBI9gBaebbnrfifHhDYfgasaacH8akY=wiFfYdH8Gipec8Eeeu0xXdbba9frFj0=OqFfea0dXdd9vqai=hGuQ8kuc9pgc9s8qqaq=dirpe0xb9q8qiLsFr0=vr0=vr0dc8meaabaqaciaacaGaaeqabaqabeGadaaakeaacqWGmbatcqGH9aqpdaqeqbqaaiabdchaWjabcIcaOiqbdogaJzaalaGaeiilaWIaemiDaqNaeiykaKYaaWbaaSqabeaacqWGUbGBcqGGOaakcuWGJbWygaWcaiabcYcaSiabdsha0jabcMcaPaaaaeaacuWGJbWygaWcaiabcYcaSiabdsha0bqab0Gaey4dIunakiabc6caUiaaxMaacaWLjaWaaeWaaeaacqaIZaWmaiaawIcacaGLPaaaaaa@46FE@

**Figure 8:**
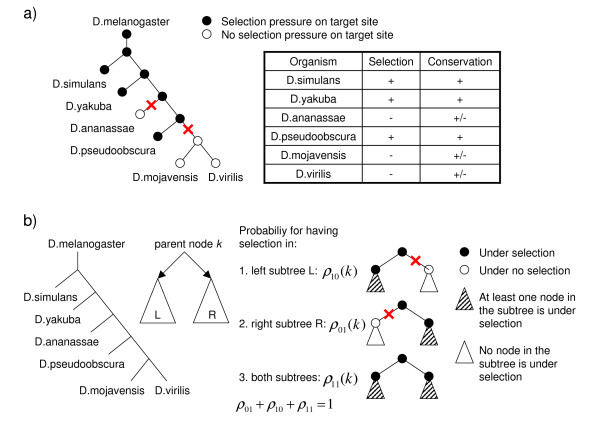
**Modeling the selection pressure on miRNA target sites**. a. The phylogenetic tree of the species in the clade (here flies) is rooted at the reference species (here melanogaster) and selection is modeled starting from the root and moving down the tree (see Methods for details). At each internal node *k *there are probabilities for selection to be maintained in one or both children of the node (see Methods for details). b. Relationship between selection and conservation patterns: Example of a selection pattern on a particular set of orthologous target sites in flies. Open circles indicate absence of selection pressure, closed circles indicate presence of selection pressure. Selection pressure is absent in Drosophila ananassae, mojavensis and virilis (D.ananassae, D.mojavensis, and D.virilis). The possible conservation patterns consistent with the selection pattern for this target site are listed in the table. The site needs to be conserved in all species in which selection pressure operates, namely Drosophila simulans, yakuba and pseudoobscura (D.simulans, D.yakuba, D.Pseudoobscura). In the species in which selection pressure does not operate, the site may or may not be conserved.

Given sufficient data, i.e. *n*(c→
 MathType@MTEF@5@5@+=feaafiart1ev1aaatCvAUfKttLearuWrP9MDH5MBPbIqV92AaeXatLxBI9gBaebbnrfifHhDYfgasaacH8akY=wiFfYdH8Gipec8Eeeu0xXdbba9frFj0=OqFfea0dXdd9vqai=hGuQ8kuc9pgc9s8qqaq=dirpe0xb9q8qiLsFr0=vr0=vr0dc8meaabaqaciaacaGaaeqabaqabeGadaaakeaacuWGJbWygaWcaaaa@2E0D@, *t*) >> 0 for all c→
 MathType@MTEF@5@5@+=feaafiart1ev1aaatCvAUfKttLearuWrP9MDH5MBPbIqV92AaeXatLxBI9gBaebbnrfifHhDYfgasaacH8akY=wiFfYdH8Gipec8Eeeu0xXdbba9frFj0=OqFfea0dXdd9vqai=hGuQ8kuc9pgc9s8qqaq=dirpe0xb9q8qiLsFr0=vr0=vr0dc8meaabaqaciaacaGaaeqabaqabeGadaaakeaacuWGJbWygaWcaaaa@2E0D@, we could estimate *p*(s→
 MathType@MTEF@5@5@+=feaafiart1ev1aaatCvAUfKttLearuWrP9MDH5MBPbIqV92AaeXatLxBI9gBaebbnrfifHhDYfgasaacH8akY=wiFfYdH8Gipec8Eeeu0xXdbba9frFj0=OqFfea0dXdd9vqai=hGuQ8kuc9pgc9s8qqaq=dirpe0xb9q8qiLsFr0=vr0=vr0dc8meaabaqaciaacaGaaeqabaqabeGadaaakeaacuWGZbWCgaWcaaaa@2E2D@) by maximizing *L *with respect to *p*(s→
 MathType@MTEF@5@5@+=feaafiart1ev1aaatCvAUfKttLearuWrP9MDH5MBPbIqV92AaeXatLxBI9gBaebbnrfifHhDYfgasaacH8akY=wiFfYdH8Gipec8Eeeu0xXdbba9frFj0=OqFfea0dXdd9vqai=hGuQ8kuc9pgc9s8qqaq=dirpe0xb9q8qiLsFr0=vr0=vr0dc8meaabaqaciaacaGaaeqabaqabeGadaaakeaacuWGZbWCgaWcaaaa@2E2D@). The amount of data is limited, however, and the distribution *p*(s→
 MathType@MTEF@5@5@+=feaafiart1ev1aaatCvAUfKttLearuWrP9MDH5MBPbIqV92AaeXatLxBI9gBaebbnrfifHhDYfgasaacH8akY=wiFfYdH8Gipec8Eeeu0xXdbba9frFj0=OqFfea0dXdd9vqai=hGuQ8kuc9pgc9s8qqaq=dirpe0xb9q8qiLsFr0=vr0=vr0dc8meaabaqaciaacaGaaeqabaqabeGadaaakeaacuWGZbWCgaWcaaaa@2E2D@) generally has a large number of independent components (2^*g *^for *g *species). As we believe that it is not possible to robustly fit the entire distribution *p*(s→
 MathType@MTEF@5@5@+=feaafiart1ev1aaatCvAUfKttLearuWrP9MDH5MBPbIqV92AaeXatLxBI9gBaebbnrfifHhDYfgasaacH8akY=wiFfYdH8Gipec8Eeeu0xXdbba9frFj0=OqFfea0dXdd9vqai=hGuQ8kuc9pgc9s8qqaq=dirpe0xb9q8qiLsFr0=vr0=vr0dc8meaabaqaciaacaGaaeqabaqabeGadaaakeaacuWGZbWCgaWcaaaa@2E2D@) without a significant risk of over-fitting, we instead aimed to parametrize reasonable distributions *p*(s→
 MathType@MTEF@5@5@+=feaafiart1ev1aaatCvAUfKttLearuWrP9MDH5MBPbIqV92AaeXatLxBI9gBaebbnrfifHhDYfgasaacH8akY=wiFfYdH8Gipec8Eeeu0xXdbba9frFj0=OqFfea0dXdd9vqai=hGuQ8kuc9pgc9s8qqaq=dirpe0xb9q8qiLsFr0=vr0=vr0dc8meaabaqaciaacaGaaeqabaqabeGadaaakeaacuWGZbWCgaWcaaaa@2E2D@) using a much smaller set of parameters, i.e. on the order of *g *rather than 2^*g *^parameters. A second piece of information that can help us estimate *p*(s→
 MathType@MTEF@5@5@+=feaafiart1ev1aaatCvAUfKttLearuWrP9MDH5MBPbIqV92AaeXatLxBI9gBaebbnrfifHhDYfgasaacH8akY=wiFfYdH8Gipec8Eeeu0xXdbba9frFj0=OqFfea0dXdd9vqai=hGuQ8kuc9pgc9s8qqaq=dirpe0xb9q8qiLsFr0=vr0=vr0dc8meaabaqaciaacaGaaeqabaqabeGadaaakeaacuWGZbWCgaWcaaaa@2E2D@) consists of the phylogenetic relationships between the species. That is, one would generally expect that functional target sites in the reference species are more often also functional in closely related species than they are in distantly related ones. It is thus natural to model the evolution of selection patterns along the branches of the phylogenetic tree of the clade. In analogy with evolutionary models for the evolution of gene sequences one might consider models in which selection for a site may "mutate" from "on" to "off" along each branch of the tree, with a probability of "mutation" proportional to the length of the branch. However, in contrast to such simple evolutionary events as point mutations in sequences, the "mutations" in our model correspond to changes in selection pressures and we see no reason to assume that these occur at a constant rate along each branch of the phylogenetic tree. Indeed, as we will see below, our results suggest that the rate of turnover of selection along a given branch of the tree differs significantly between miRNAs. To reasonable parametrize *p*(s→
 MathType@MTEF@5@5@+=feaafiart1ev1aaatCvAUfKttLearuWrP9MDH5MBPbIqV92AaeXatLxBI9gBaebbnrfifHhDYfgasaacH8akY=wiFfYdH8Gipec8Eeeu0xXdbba9frFj0=OqFfea0dXdd9vqai=hGuQ8kuc9pgc9s8qqaq=dirpe0xb9q8qiLsFr0=vr0=vr0dc8meaabaqaciaacaGaaeqabaqabeGadaaakeaacuWGZbWCgaWcaaaa@2E2D@) we would therefore have to fit independent rates of loss and gain of selection along each branch of the tree for each miRNA. In addition, for every selection pattern s→
 MathType@MTEF@5@5@+=feaafiart1ev1aaatCvAUfKttLearuWrP9MDH5MBPbIqV92AaeXatLxBI9gBaebbnrfifHhDYfgasaacH8akY=wiFfYdH8Gipec8Eeeu0xXdbba9frFj0=OqFfea0dXdd9vqai=hGuQ8kuc9pgc9s8qqaq=dirpe0xb9q8qiLsFr0=vr0=vr0dc8meaabaqaciaacaGaaeqabaqabeGadaaakeaacuWGZbWCgaWcaaaa@2E2D@ we would need to consider all evolutionary histories of selection loss and gain that are consistent with the resulting selection pattern at the leaves of the tree. Finally, note that we inherently treat the species in the clade asymmetrically. That is, we look for putative sites in the reference species only and then use pairwise genome alignments to determine the conservation pattern of each putative site in the reference species. We thus by definition never consider conservation patterns in which the site is conserved in some of the species but *not *in the reference species. In summary, we looked for a parametrization of *p*(s→
 MathType@MTEF@5@5@+=feaafiart1ev1aaatCvAUfKttLearuWrP9MDH5MBPbIqV92AaeXatLxBI9gBaebbnrfifHhDYfgasaacH8akY=wiFfYdH8Gipec8Eeeu0xXdbba9frFj0=OqFfea0dXdd9vqai=hGuQ8kuc9pgc9s8qqaq=dirpe0xb9q8qiLsFr0=vr0=vr0dc8meaabaqaciaacaGaaeqabaqabeGadaaakeaacuWGZbWCgaWcaaaa@2E2D@) that is flexible enough to allow for different rates of turnover of selection along each branch of the tree, that respects the topology of the phylogenetic tree, that takes into account our inherent asymmetric treatment of the reference species, and that minimizes the number of free parameters needed, so that over-fitting is avoided as much as possible. The parametrization that we chose is the following. We take the phylogenetic tree of the set of related species, and take the reference species as the root of the tree, as illustrated in Figure 8a for the Drosophila species. Starting from a functional site in the reference species we now move along the tree from top to bottom and assume that in each branch the "functionality" of the site can only be *lost*. That is, if the site is not under selection at a given internal node of the tree, we assume that it is also not under selection in any of its descendants. The probabilities *p*(s→
 MathType@MTEF@5@5@+=feaafiart1ev1aaatCvAUfKttLearuWrP9MDH5MBPbIqV92AaeXatLxBI9gBaebbnrfifHhDYfgasaacH8akY=wiFfYdH8Gipec8Eeeu0xXdbba9frFj0=OqFfea0dXdd9vqai=hGuQ8kuc9pgc9s8qqaq=dirpe0xb9q8qiLsFr0=vr0=vr0dc8meaabaqaciaacaGaaeqabaqabeGadaaakeaacuWGZbWCgaWcaaaa@2E2D@) can then be parametrized by giving, at each node *k*, the probabilities *ρ*_11_(*k*), *ρ*_10_(*k*) and *ρ*_01_(*k*) that the functionality is maintained in both descendants, in the left descendant only, or the right descendant only (Figure 8b). Note that we assume that if the site was not under selection in either descendant then the site was already not under selection in the parent, and that at each node *k *the probabilities sum to one, *ρ*_11_(*k*) + *ρ*_10_(*k*) + *ρ*_01_(*k*) = 1. There are thus 10 independent parameters for the Drosophila tree of Figure 8a which has 5 internal nodes. A final parameter *ρ *gives the probability that functionality is maintained in going from the reference species to the first internal node. Thus, with probability *ρ *the site is conserved in at least one of the other species, and with probability (1 - *ρ*) it is specific to the reference species. The tree in Figure 8a shows a selection pattern with selection in D. simulans, D. yakuba and D. pseudoobscura. Using our parametrization the prior probability of this selection pattern is *ρρ*_11_(1)*ρ*_11_(2)*ρ*_01_(3)*ρ*_10_(4) (we number the nodes from top to bottom). A nice feature of this parametrization of *p*(s→
 MathType@MTEF@5@5@+=feaafiart1ev1aaatCvAUfKttLearuWrP9MDH5MBPbIqV92AaeXatLxBI9gBaebbnrfifHhDYfgasaacH8akY=wiFfYdH8Gipec8Eeeu0xXdbba9frFj0=OqFfea0dXdd9vqai=hGuQ8kuc9pgc9s8qqaq=dirpe0xb9q8qiLsFr0=vr0=vr0dc8meaabaqaciaacaGaaeqabaqabeGadaaakeaacuWGZbWCgaWcaaaa@2E2D@) is that the selection at all internal nodes of the tree is uniquely determined by the selection at the leaves of the tree, i.e. no sum over different evolutionary histories is required.

Note that by using only conservation information, we cannot possibly distinguish sites that are only functional in the reference species from sites that are not functional at all. That is, we do not know what part of the fraction (1 - *ρ*) corresponds to sites that are functional, reference-specific sites and what fraction is nonfunctional. The inferred fraction *ρ *therefore provides a *lower bound *on the fraction of functional sites. For simplicity, we will make the conservative assumption that only the fraction *ρ *of sites is functional, and refer to these sites as the fraction of "functional" sites.

For each miRNA we estimate the parameters *ρ*, and *ρ*_11_(*k*), *ρ*_10_(*k*) and *ρ*_01_(*k*) for each node *k*, by maximizing the likelihood of the distribution given the observed data, i.e. equation (3). Let *ω *denote one of the possible selection patterns for the two descending branches, i.e. *ω *∈ {01, 10, 11}, and define the indicator function *δ*(s→
 MathType@MTEF@5@5@+=feaafiart1ev1aaatCvAUfKttLearuWrP9MDH5MBPbIqV92AaeXatLxBI9gBaebbnrfifHhDYfgasaacH8akY=wiFfYdH8Gipec8Eeeu0xXdbba9frFj0=OqFfea0dXdd9vqai=hGuQ8kuc9pgc9s8qqaq=dirpe0xb9q8qiLsFr0=vr0=vr0dc8meaabaqaciaacaGaaeqabaqabeGadaaakeaacuWGZbWCgaWcaaaa@2E2D@, *ω*, *k*) such that *δ *(s→
 MathType@MTEF@5@5@+=feaafiart1ev1aaatCvAUfKttLearuWrP9MDH5MBPbIqV92AaeXatLxBI9gBaebbnrfifHhDYfgasaacH8akY=wiFfYdH8Gipec8Eeeu0xXdbba9frFj0=OqFfea0dXdd9vqai=hGuQ8kuc9pgc9s8qqaq=dirpe0xb9q8qiLsFr0=vr0=vr0dc8meaabaqaciaacaGaaeqabaqabeGadaaakeaacuWGZbWCgaWcaaaa@2E2D@, *ω*, *k*) = 1 whenever the parameter *ρ*_*ω*_(*k*) occurs in *p*(s→
 MathType@MTEF@5@5@+=feaafiart1ev1aaatCvAUfKttLearuWrP9MDH5MBPbIqV92AaeXatLxBI9gBaebbnrfifHhDYfgasaacH8akY=wiFfYdH8Gipec8Eeeu0xXdbba9frFj0=OqFfea0dXdd9vqai=hGuQ8kuc9pgc9s8qqaq=dirpe0xb9q8qiLsFr0=vr0=vr0dc8meaabaqaciaacaGaaeqabaqabeGadaaakeaacuWGZbWCgaWcaaaa@2E2D@) and *δ *(s→
 MathType@MTEF@5@5@+=feaafiart1ev1aaatCvAUfKttLearuWrP9MDH5MBPbIqV92AaeXatLxBI9gBaebbnrfifHhDYfgasaacH8akY=wiFfYdH8Gipec8Eeeu0xXdbba9frFj0=OqFfea0dXdd9vqai=hGuQ8kuc9pgc9s8qqaq=dirpe0xb9q8qiLsFr0=vr0=vr0dc8meaabaqaciaacaGaaeqabaqabeGadaaakeaacuWGZbWCgaWcaaaa@2E2D@, *ω*, *k*) = 0 when it does not. We then have for the derivatives

dp(s→)dρω(k)=δ(s→,ω,k)p(s→)ρω(k).     (4)
 MathType@MTEF@5@5@+=feaafiart1ev1aaatCvAUfKttLearuWrP9MDH5MBPbIqV92AaeXatLxBI9gBaebbnrfifHhDYfgasaacH8akY=wiFfYdH8Gipec8Eeeu0xXdbba9frFj0=OqFfea0dXdd9vqai=hGuQ8kuc9pgc9s8qqaq=dirpe0xb9q8qiLsFr0=vr0=vr0dc8meaabaqaciaacaGaaeqabaqabeGadaaakeaadaWcaaqaaiabdsgaKjabdchaWjabcIcaOiqbdohaZzaalaGaeiykaKcabaGaemizaqgcciGae8xWdi3aaSbaaSqaaiab=L8a3bqabaGccqGGOaakcqWGRbWAcqGGPaqkaaGaeyypa0Jae8hTdqMaeiikaGIafm4CamNbaSaacqGGSaalcqWFjpWDcqGGSaalcqWGRbWAcqGGPaqkdaWcaaqaaiabdchaWjabcIcaOiqbdohaZzaalaGaeiykaKcabaGae8xWdi3aaSbaaSqaaiab=L8a3bqabaGccqGGOaakcqWGRbWAcqGGPaqkaaGaeiOla4IaaCzcaiaaxMaadaqadaqaaiabisda0aGaayjkaiaawMcaaaaa@55AD@

Using this it is easy to show that *L *can be maximized with respect to the parameters *ρ*_*ω*_(*k*) by an expectation maximization (EM) procedure. If we define

Xω(k)=∑c→,tn(c→,t)[∑s→∈Sδ(s→,ω,k)p(c→|t,s→)p(s→)∑σ→∈Sp(c→|t,σ→)p(σ→)]     (5)
 MathType@MTEF@5@5@+=feaafiart1ev1aaatCvAUfKttLearuWrP9MDH5MBPbIqV92AaeXatLxBI9gBaebbnrfifHhDYfgasaacH8akY=wiFfYdH8Gipec8Eeeu0xXdbba9frFj0=OqFfea0dXdd9vqai=hGuQ8kuc9pgc9s8qqaq=dirpe0xb9q8qiLsFr0=vr0=vr0dc8meaabaqaciaacaGaaeqabaqabeGadaaakeaacqWGybawdaWgaaWcbaacciGae8xYdChabeaakiabcIcaOiabdUgaRjabcMcaPiabg2da9maaqafabaGaemOBa4MaeiikaGIafm4yamMbaSaacqGGSaalcqWG0baDcqGGPaqkdaWadaqaamaaqafabaGae8hTdqMaeiikaGIafm4CamNbaSaacqGGSaalcqWFjpWDcqGGSaalcqWGRbWAcqGGPaqkdaWcaaqaaiabdchaWjabcIcaOiqbdogaJzaalaGaeiiFaWNaemiDaqNaeiilaWIafm4CamNbaSaacqGGPaqkcqWGWbaCcqGGOaakcuWGZbWCgaWcaiabcMcaPaqaamaaqababaGaemiCaaNaeiikaGIafm4yamMbaSaacqGG8baFcqWG0baDcqGGSaalcuWFdpWCgaWcaiabcMcaPiabdchaWjabcIcaOiqb=n8aZzaalaGaeiykaKcaleaacuWFdpWCgaWcaiabgIGiolabdofatbqab0GaeyyeIuoaaaaaleaacuWGZbWCgaWcaiabgIGiolabdofatbqab0GaeyyeIuoaaOGaay5waiaaw2faaaWcbaGafm4yamMbaSaacqGGSaalcqWG0baDaeqaniabggHiLdGccaWLjaGaaCzcamaabmaabaGaeGynaudacaGLOaGaayzkaaaaaa@7A68@

then the EM update equations are given by

ρω(k)=Xω(k)∑ω˜∈{01,10,11}Xω˜(k).     (6)
 MathType@MTEF@5@5@+=feaafiart1ev1aaatCvAUfKttLearuWrP9MDH5MBPbIqV92AaeXatLxBI9gBaebbnrfifHhDYfgasaacH8akY=wiFfYdH8Gipec8Eeeu0xXdbba9frFj0=OqFfea0dXdd9vqai=hGuQ8kuc9pgc9s8qqaq=dirpe0xb9q8qiLsFr0=vr0=vr0dc8meaabaqaciaacaGaaeqabaqabeGadaaakeaaiiGacqWFbpGCdaWgaaWcbaGae8xYdChabeaakiabcIcaOiabdUgaRjabcMcaPiabg2da9maalaaabaGaemiwaG1aaSbaaSqaaiab=L8a3bqabaGccqGGOaakcqWGRbWAcqGGPaqkaeaadaaeqaqaaiabdIfaynaaBaaaleaacuWFjpWDgaacaiabcIcaOiabdUgaRjabcMcaPaqabaaabaGaf8xYdCNbaGaacqGHiiIZcqGG7bWEcqaIWaamcqaIXaqmcqGGSaalcqaIXaqmcqaIWaamcqGGSaalcqaIXaqmcqaIXaqmcqGG9bqFaeqaniabggHiLdaaaOGaeiOla4IaaCzcaiaaxMaadaqadaqaaiabiAda2aGaayjkaiaawMcaaaaa@5571@

By iterating these equations we can determine the optimal *ρ*_*ω*_(*k*). Since, as can also be shown by taking second derivatives, the likelihood *L *is a concave function of the parameters *ρ*_*ω*_(*k*), the EM procedure is guaranteed to converge to the unique global optimum of the likelihood.

Once all *ρ*_*ω*_(*k*) have been determined for a given miRNA we can calculate posterior probabilities of functionality for each putative target site as follows. As mentioned above, we consider a target site functional if it is under selection in the reference and at least one other species. The nonfunctional sites are then by definition those sites that are not under selection in any of the other species. We will denote this no-selection pattern as 0→
 MathType@MTEF@5@5@+=feaafiart1ev1aaatCvAUfKttLearuWrP9MDH5MBPbIqV92AaeXatLxBI9gBaebbnrfifHhDYfgasaacH8akY=wiFfYdH8Gipec8Eeeu0xXdbba9frFj0=OqFfea0dXdd9vqai=hGuQ8kuc9pgc9s8qqaq=dirpe0xb9q8qiLsFr0=vr0=vr0dc8meaabaqaciaacaGaaeqabaqabeGadaaakeaacuaIWaamgaWcaaaa@2DAC@. For a site of seed type *t *and conservation pattern c→
 MathType@MTEF@5@5@+=feaafiart1ev1aaatCvAUfKttLearuWrP9MDH5MBPbIqV92AaeXatLxBI9gBaebbnrfifHhDYfgasaacH8akY=wiFfYdH8Gipec8Eeeu0xXdbba9frFj0=OqFfea0dXdd9vqai=hGuQ8kuc9pgc9s8qqaq=dirpe0xb9q8qiLsFr0=vr0=vr0dc8meaabaqaciaacaGaaeqabaqabeGadaaakeaacuWGJbWygaWcaaaa@2E0D@ the posterior probability that the site is functional is then given by

p(s→≠0→|t,c→)=1−p(c→|t,0→)p(0→)∑s→∈Sp(c→|t,s→)p(s→).     (7)
 MathType@MTEF@5@5@+=feaafiart1ev1aaatCvAUfKttLearuWrP9MDH5MBPbIqV92AaeXatLxBI9gBaebbnrfifHhDYfgasaacH8akY=wiFfYdH8Gipec8Eeeu0xXdbba9frFj0=OqFfea0dXdd9vqai=hGuQ8kuc9pgc9s8qqaq=dirpe0xb9q8qiLsFr0=vr0=vr0dc8meaabaqaciaacaGaaeqabaqabeGadaaakeaacqWGWbaCcqGGOaakcuWGZbWCgaWcaiabgcMi5kqbicdaWyaalaGaeiiFaWNaemiDaqNaeiilaWIafm4yamMbaSaacqGGPaqkcqGH9aqpcqaIXaqmcqGHsisldaWcaaqaaiabdchaWjabcIcaOiqbdogaJzaalaGaeiiFaWNaemiDaqNaeiilaWIafGimaaJbaSaacqGGPaqkcqWGWbaCcqGGOaakcuaIWaamgaWcaiabcMcaPaqaamaaqababaGaemiCaaNaeiikaGIafm4yamMbaSaacqGG8baFcqWG0baDcqGGSaalcuWGZbWCgaWcaiabcMcaPiabdchaWjabcIcaOiqbdohaZzaalaGaeiykaKcaleaacuWGZbWCgaWcaiabgIGiolabdofatbqab0GaeyyeIuoaaaGccqGGUaGlcaWLjaGaaCzcamaabmaabaGaeG4naCdacaGLOaGaayzkaaaaaa@62D4@

Note that the prior probability of no selection is simply (1 - *ρ*), i.e. *p*(0→
 MathType@MTEF@5@5@+=feaafiart1ev1aaatCvAUfKttLearuWrP9MDH5MBPbIqV92AaeXatLxBI9gBaebbnrfifHhDYfgasaacH8akY=wiFfYdH8Gipec8Eeeu0xXdbba9frFj0=OqFfea0dXdd9vqai=hGuQ8kuc9pgc9s8qqaq=dirpe0xb9q8qiLsFr0=vr0=vr0dc8meaabaqaciaacaGaaeqabaqabeGadaaakeaacuaIWaamgaWcaaaa@2DAC@) = 1 - *ρ*, and that the probability for conservation pattern c→
 MathType@MTEF@5@5@+=feaafiart1ev1aaatCvAUfKttLearuWrP9MDH5MBPbIqV92AaeXatLxBI9gBaebbnrfifHhDYfgasaacH8akY=wiFfYdH8Gipec8Eeeu0xXdbba9frFj0=OqFfea0dXdd9vqai=hGuQ8kuc9pgc9s8qqaq=dirpe0xb9q8qiLsFr0=vr0=vr0dc8meaabaqaciaacaGaaeqabaqabeGadaaakeaacuWGJbWygaWcaaaa@2E0D@ given no selection is simply the background probability

p(c→|t,0→)=p(c→|t,bg).     (8)
 MathType@MTEF@5@5@+=feaafiart1ev1aaatCvAUfKttLearuWrP9MDH5MBPbIqV92AaeXatLxBI9gBaebbnrfifHhDYfgasaacH8akY=wiFfYdH8Gipec8Eeeu0xXdbba9frFj0=OqFfea0dXdd9vqai=hGuQ8kuc9pgc9s8qqaq=dirpe0xb9q8qiLsFr0=vr0=vr0dc8meaabaqaciaacaGaaeqabaqabeGadaaakeaacqWGWbaCcqGGOaakcuWGJbWygaWcaiabcYha8jabdsha0jabcYcaSiqbicdaWyaalaGaeiykaKIaeyypa0JaemiCaaNaeiikaGIafm4yamMbaSaacqGG8baFcqWG0baDcqGGSaalcqqGIbGycqqGNbWzcqGGPaqkcqGGUaGlcaWLjaGaaCzcamaabmaabaGaeGioaGdacaGLOaGaayzkaaaaaa@469B@

We can thus also write the posterior as

p(s→≠0→|t,c→)=1−p(c→|t,bg)(1−ρ)∑s→∈Sp(c→|t,s→)p(s→).     (9)
 MathType@MTEF@5@5@+=feaafiart1ev1aaatCvAUfKttLearuWrP9MDH5MBPbIqV92AaeXatLxBI9gBaebbnrfifHhDYfgasaacH8akY=wiFfYdH8Gipec8Eeeu0xXdbba9frFj0=OqFfea0dXdd9vqai=hGuQ8kuc9pgc9s8qqaq=dirpe0xb9q8qiLsFr0=vr0=vr0dc8meaabaqaciaacaGaaeqabaqabeGadaaakeaacqWGWbaCcqGGOaakcuWGZbWCgaWcaiabgcMi5kqbicdaWyaalaGaeiiFaWNaemiDaqNaeiilaWIafm4yamMbaSaacqGGPaqkcqGH9aqpcqaIXaqmcqGHsisldaWcaaqaaiabdchaWjabcIcaOiqbdogaJzaalaGaeiiFaWNaemiDaqNaeiilaWIaeeOyaiMaee4zaCMaeiykaKIaeiikaGIaeGymaeJaeyOeI0ccciGae8xWdiNaeiykaKcabaWaaabeaeaacqWGWbaCcqGGOaakcuWGJbWygaWcaiabcYha8jabdsha0jabcYcaSiqbdohaZzaalaGaeiykaKIaemiCaaNaeiikaGIafm4CamNbaSaacqGGPaqkaSqaaiqbdohaZzaalaGaeyicI4Saem4uamfabeqdcqGHris5aaaakiabc6caUiaaxMaacaWLjaWaaeWaaeaacqaI5aqoaiaawIcacaGLPaaaaaa@65B3@

The parameter *ρ *thus corresponds to the estimated fraction of all putative target sites in the reference that are functional, i.e. under selection in at least on other species.

Finally, note that the sums in equations (1) and (2) involve a number of terms that grows exponentially with the number of species in the clade. We believe that this will not cause any computational problems in clades with less than 20 or so species. For much larger sets of species these sums can become computationally prohibitive. In those circumstances one could reduce the number of species by choosing, for each set of closely-related species, only a single representative. For species that are so closely-related that most putative target sites are conserved between them, choosing a single representative per group would hardly affect the predictions.

### Sequence data

We carried out miRNA target predictions for all available human, fly, fish and worm RefSeq transcripts present in the 17th release of the Refseq database. We mapped all transcripts to the corresponding genomes using the Spa cDNA-to-genome alignment program [65], and the genome assemblies hg17 (human), dm2 (fly), ceWB05 (worm) and danRer3 (fish) provided by the Genome Bioinformatics group at the University of California, Santa Cruz [66]. From the same source we also downloaded pairwise alignments of several genomes with the genome of reference species, as follows: for human we downloaded hg17-to-panTro1, hg17-to-rheMac2, hg17-to-canFam2, hg17-to-bosTau2, hg17-to-mm7, hg17-to-rn3, hg17-to-monDom1 and hg17-to-galGal2; for fly we used dm2-to-droSim1, dm2-to-droYak1, dm2-to-droAna1, dm2-to-dp3, dm2-to-droMoj1, dm2-to-droVir1; for fish we used danRer3-to-fr1 and danRer3-to-tetNig1. Finally, for worm we used the software Threaded Blockset Aligner (TBA) [67] to align C. briggsae and C. remanei to C. elegans.

### Pathway enrichment analysis

We used the KEGG database to infer pathways preferentially targeted by individual miRNAs. The KEGG database (ftp.genome.jp) contains mappings from NCBI Gene identifiers to pathway IDs (data files: [org]_ncbi-geneid.list, with [org] being the species code)), while the Gene database of NCBI () provides mappings from Gene IDs to Refseq IDs (gene2refseq). By intersecting these data sets we obtained the mappings from Refseq IDs to pathways. We then used a Bayesian method to determine the significance of the overlap between the targets of each seed-equivalent set of miRNAs and each specific pathway.

For a given pathway and miRNA let *n*_01_, *n*_10_, *n*_00 _and *n*_11 _denote respectively the number of predicted targets of the miRNA that are not part of the pathway, the number of genes in the pathway that are not targeted by the miRNA, the number of genes that are neither targets of the miRNA nor members of the pathway, and the number of genes in the pathway that are predicted to be targeted by the miRNA. While pathway membership is a simple boolean variable (a gene is either a member of a given pathway or it is not), we can only assign probabilities for a given gene to be a miRNA target. Assume that a given gene has *n *putative target sites for a given miRNA and let *p*_*i *_denote the posterior probability of the *i*th site. The probability that at least one of the sites is functional is then given by ptar=1−∏i=1n(1−pi)
MathType@MTEF@5@5@+=feaafiart1ev1aaatCvAUfKttLearuWrP9MDH5MBPbIqV92AaeXatLxBI9gBaebbnrfifHhDYfgasaacH8akY=wiFfYdH8Gipec8Eeeu0xXdbba9frFj0=OqFfea0dXdd9vqai=hGuQ8kuc9pgc9s8qqaq=dirpe0xb9q8qiLsFr0=vr0=vr0dc8meaabaqaciaacaGaaeqabaqabeGadaaakeaacqWGWbaCdaWgaaWcbaGaeeiDaqNaeeyyaeMaeeOCaihabeaakiabg2da9iabigdaXiabgkHiTmaaradabaGaeiikaGIaeGymaeJaeyOeI0IaemiCaa3aaSbaaSqaaiabdMgaPbqabaGccqGGPaqkaSqaaiabdMgaPjabg2da9iabigdaXaqaaiabd6gaUbqdcqGHpis1aaaa@4281@. We use *p*_tar _as the probability that the gene is targeted by the miRNA and obtain *n*_01 _and *n*_11 _by summing *p*_tar _over all genes that are not in the pathway and all genes in the pathway respectively. Similarly we sum (1 - *p*_tar_) over all genes that are not in the pathway and all genes in the pathway to obtain *n*_00 _and *n*_10 _respectively. Finally we calculate the probability of the observed counts *n*_00_, *n*_10_, *n*_01_, and *n*_11 _under an "independent model", in which the probability to be targeted by the miRNA is independent of pathway membership, and a "dependent model" in which the probability of miRNA targeting is generally dependent on pathway membership. The likelihood under the independent model is given by

Lindep=∫01(pq)n11(p(1−q))n10((1−p)q)n01((1−p)(1−q))n00dpdq=Γ(n1.+1)Γ(n0.+1)Γ(n.0+1)Γ(n.1+1)Γ(n+2)Γ(n+2),     (10)
 MathType@MTEF@5@5@+=feaafiart1ev1aaatCvAUfKttLearuWrP9MDH5MBPbIqV92AaeXatLxBI9gBaebbnrfifHhDYfgasaacH8akY=wiFfYdH8Gipec8Eeeu0xXdbba9frFj0=OqFfea0dXdd9vqai=hGuQ8kuc9pgc9s8qqaq=dirpe0xb9q8qiLsFr0=vr0=vr0dc8meaabaqaciaacaGaaeqabaqabeGadaaakeaafaqaaeWadaaabaGaemitaW0aaSbaaSqaaiabbMgaPjabb6gaUjabbsgaKjabbwgaLjabbchaWbqabaaakeaacqGH9aqpaeaadaWdXaqaaiabcIcaOiabdchaWjabdghaXjabcMcaPmaaCaaaleqabaGaemOBa42aaSbaaWqaaiabigdaXiabigdaXaqabaaaaaWcbaGaeGimaadabaGaeGymaedaniabgUIiYdGccqGGOaakcqWGWbaCcqGGOaakcqaIXaqmcqGHsislcqWGXbqCcqGGPaqkcqGGPaqkdaahaaWcbeqaaiabd6gaUnaaBaaameaacqaIXaqmcqaIWaamaeqaaaaaaOqaaaqaaaqaaiabcIcaOiabcIcaOiabigdaXiabgkHiTiabdchaWjabcMcaPiabdghaXjabcMcaPmaaCaaaleqabaGaemOBa42aaSbaaWqaaiabicdaWiabigdaXaqabaaaaOGaeiikaGIaeiikaGIaeGymaeJaeyOeI0IaemiCaaNaeiykaKIaeiikaGIaeGymaeJaeyOeI0IaemyCaeNaeiykaKIaeiykaKYaaWbaaSqabeaacqWGUbGBdaWgaaadbaGaeGimaaJaeGimaadabeaaaaGccqWGKbazcqWGWbaCcqWGKbazcqWGXbqCaeaaaeaacqGH9aqpaeaadaWcaaqaaiabfo5ahjabcIcaOiabd6gaUnaaBaaaleaacqaIXaqmcqGGUaGlaeqaaOGaey4kaSIaeGymaeJaeiykaKIaeu4KdCKaeiikaGIaemOBa42aaSbaaSqaaiabicdaWiabc6caUaqabaGccqGHRaWkcqaIXaqmcqGGPaqkcqqHtoWrcqGGOaakcqWGUbGBdaWgaaWcbaGaeiOla4IaeGimaadabeaakiabgUcaRiabigdaXiabcMcaPiabfo5ahjabcIcaOiabd6gaUnaaBaaaleaacqGGUaGlcqaIXaqmaeqaaOGaey4kaSIaeGymaeJaeiykaKcabaGaeu4KdCKaeiikaGIaemOBa4Maey4kaSIaeGOmaiJaeiykaKIaeu4KdCKaeiikaGIaemOBa4Maey4kaSIaeGOmaiJaeiykaKcaaiabcYcaSaaacaWLjaGaaCzcamaabmaabaGaeGymaeJaeGimaadacaGLOaGaayzkaaaaaa@A2FE@

where Γ(*x*) is the gamma function, a dot indicates summation over the variable in question, i.e. *n*_1. _= *n*_10 _+ *n*_11 _and *n *is the total number of genes. For the dependent model the likelihood is given by

Ldep=∫p00n00p10n10p01n01p11n11dp00dp01dp10dp11=Γ(4)Γ(n11+1)Γ(n10+1)Γ(n01+1)Γ(n00+1)Γ(n+4),     (11)
 MathType@MTEF@5@5@+=feaafiart1ev1aaatCvAUfKttLearuWrP9MDH5MBPbIqV92AaeXatLxBI9gBaebbnrfifHhDYfgasaacH8akY=wiFfYdH8Gipec8Eeeu0xXdbba9frFj0=OqFfea0dXdd9vqai=hGuQ8kuc9pgc9s8qqaq=dirpe0xb9q8qiLsFr0=vr0=vr0dc8meaabaqaciaacaGaaeqabaqabeGadaaakeaafaqaaeGadaaabaGaemitaW0aaSbaaSqaaiabbsgaKjabbwgaLjabbchaWbqabaaakeaacqGH9aqpaeaadaWdbaqaaiabdchaWnaaDaaaleaacqaIWaamcqaIWaamaeaacqWGUbGBdaWgaaadbaGaeGimaaJaeGimaadabeaaaaGccqWGWbaCdaqhaaWcbaGaeGymaeJaeGimaadabaGaemOBa42aaSbaaWqaaiabigdaXiabicdaWaqabaaaaOGaemiCaa3aa0baaSqaaiabicdaWiabigdaXaqaaiabd6gaUnaaBaaameaacqaIWaamcqaIXaqmaeqaaaaakiabdchaWnaaDaaaleaacqaIXaqmcqaIXaqmaeaacqWGUbGBdaWgaaadbaGaeGymaeJaeGymaedabeaaaaGccqWGKbazcqWGWbaCdaWgaaWcbaGaeGimaaJaeGimaadabeaakiabdsgaKjabdchaWnaaBaaaleaacqaIWaamcqaIXaqmaeqaaOGaemizaqMaemiCaa3aaSbaaSqaaiabigdaXiabicdaWaqabaGccqWGKbazcqWGWbaCdaWgaaWcbaGaeGymaeJaeGymaedabeaaaeqabeqdcqGHRiI8aaGcbaaabaGaeyypa0dabaWaaSaaaeaacqqHtoWrcqGGOaakcqaI0aancqGGPaqkcqqHtoWrcqGGOaakcqWGUbGBdaWgaaWcbaGaeGymaeJaeGymaedabeaakiabgUcaRiabigdaXiabcMcaPiabfo5ahjabcIcaOiabd6gaUnaaBaaaleaacqaIXaqmcqaIWaamaeqaaOGaey4kaSIaeGymaeJaeiykaKIaeu4KdCKaeiikaGIaemOBa42aaSbaaSqaaiabicdaWiabigdaXaqabaGccqGHRaWkcqaIXaqmcqGGPaqkcqqHtoWrcqGGOaakcqWGUbGBdaWgaaWcbaGaeGimaaJaeGimaadabeaakiabgUcaRiabigdaXiabcMcaPaqaaiabfo5ahjabcIcaOiabd6gaUjabgUcaRiabisda0iabcMcaPaaacqGGSaalaaGaaCzcaiaaxMaadaqadaqaaiabigdaXiabigdaXaGaayjkaiaawMcaaaaa@96A0@

where the integral is over the simplex *p*_00 _+ *p*_10 _+ *p*_01 _+ *p*_11 _= 1. The ratio of likelihoods *L*_dep_/*L*_indep _quantifies the amount of evidence for association between the miRNA targets and the pathway. This association can either be positive (miRNA targets are enriched in the pathway) or negative (miRNA targets are depleted in the pathway). In Figure 7 we plotted the quantity sign(*n*_11_*n*_.. _- *n*_1._*n*_.1_)*p*_dep_, where pdep=LdepLindep+Ldep
 MathType@MTEF@5@5@+=feaafiart1ev1aaatCvAUfKttLearuWrP9MDH5MBPbIqV92AaeXatLxBI9gBaebbnrfifHhDYfgasaacH8akY=wiFfYdH8Gipec8Eeeu0xXdbba9frFj0=OqFfea0dXdd9vqai=hGuQ8kuc9pgc9s8qqaq=dirpe0xb9q8qiLsFr0=vr0=vr0dc8meaabaqaciaacaGaaeqabaqabeGadaaakeaacqWGWbaCdaWgaaWcbaGaeeizaqMaeeyzauMaeeiCaahabeaakiabg2da9maalaaabaGaemitaW0aaSbaaSqaaiabbsgaKjabbwgaLjabbchaWbqabaaakeaacqWGmbatdaWgaaWcbaGaeeyAaKMaeeOBa4MaeeizaqMaeeyzauMaeeiCaahabeaakiabgUcaRiabdYeamnaaBaaaleaacqqGKbazcqqGLbqzcqqGWbaCaeqaaaaaaaa@4716@ is the posterior probability of the dependent model (assuming a uniform prior).

## Authors' contributions

DG, EvN, MZ contributed to all the stages of this project. JH implemented the web server. All authors read and approved the final manuscript.

## Additional Files

**Supplementary Data – Lists of predicted miRNA target sites**. Lists of predicted miRNA target sites in the sets of species that we studied, as well as miRNA/pathway associations are available from [68].

## Supplementary Material

Additional File 1 **Phylogenetic distribution of functional target sites**. Inferred selection pattern distributions *p*(s→
 MathType@MTEF@5@5@+=feaafiart1ev1aaatCvAUfKttLearuWrP9MDH5MBPbIqV92AaeXatLxBI9gBaebbnrfifHhDYfgasaacH8akY=wiFfYdH8Gipec8Eeeu0xXdbba9frFj0=OqFfea0dXdd9vqai=hGuQ8kuc9pgc9s8qqaq=dirpe0xb9q8qiLsFr0=vr0=vr0dc8meaabaqaciaacaGaaeqabaqabeGadaaakeaacuWGZbWCgaWcaaaa@2E2D@) for all miRNAs that are conserved in all vertebrate (panel a) and all fly (panel b) species. Each row corresponds to a miRNA seed and each column corresponds to one of the variables *ρ*_*ω*_(*k*) – where *k *indicates the internal node in the tree and *ω *indicates which of the subtrees are under selection – that parametrize *p*(s→
 MathType@MTEF@5@5@+=feaafiart1ev1aaatCvAUfKttLearuWrP9MDH5MBPbIqV92AaeXatLxBI9gBaebbnrfifHhDYfgasaacH8akY=wiFfYdH8Gipec8Eeeu0xXdbba9frFj0=OqFfea0dXdd9vqai=hGuQ8kuc9pgc9s8qqaq=dirpe0xb9q8qiLsFr0=vr0=vr0dc8meaabaqaciaacaGaaeqabaqabeGadaaakeaacuWGZbWCgaWcaaaa@2E2D@) (see Methods). The miRNAs are sorted by the inferred total fraction *ρ *of putative target sites that is under selection in at least one other species. Click here for file

Additional File 2 Number of miRNA targets predicted to be under selection pressure for each miRNA. Click here for file

Additional File 3 Detailed comparison of the overlap between the predictions provided by our method and the methods of Stark et al. [25] and Grün et al. [23]. Click here for file

Additional File 4 **Profile of the exon coverage of short (left panel) and long (right panel) 3' UTRs**. We used the mappings of spliced ESTs from the UCSC database to determine, for each nucleotide in a 3' UTR in our data set, the fraction of times the nucleotide has been observed in an exon, as opposed to an intron. We only used ESTs that mapped uniquely with at least 95% identity to the genome. Genome gaps longer than 30 nucleotides were considered to be introns. The profiles of the computed exon coverage along relatively short (less than 2 kb, left panel) and relatively long (longer than 4 kb, right panel) 3' UTRs are shown in the plots with a continuous line. Also shown are the histograms of the relative positions of predicted sites (with posterior probability ≥ 0.5) in the same 3' UTRs. Click here for file

Additional File 5 **Pathway analysis for all miRNAs and all KEGG pathways**. Representation of individual pathways among the predicted targets of a given miRNA. Each column corresponds to a KEGG pathway and each row to a group of miRNAs with the same seed sequence. Red indicates overrepresentation of the targets of a specific miRNA among the genes in the corresponding pathway, whereas blue indicates depletion. The intensity of the color indicates the posterior probability of the dependent model. Pathways have been grouped in larger functional categories according to the KEGG annotation. Click here for file

Additional File 6 **MiRNA seed families**. All miRNAs that have the same seed (positions 1–8) were clustered together. The table shows the representative miRNA as well as the members of each cluster. Click here for file
