# Supplementary figures and images for: Inference of miRNA targets using evolutionary conservation and pathway analysis
Source: BMC Bioinformatics. 2007 Mar 1;8:69. doi: 10.1186/1471-2105-8-69 (PMC1838429; doi:10.1186/1471-2105-8-69)

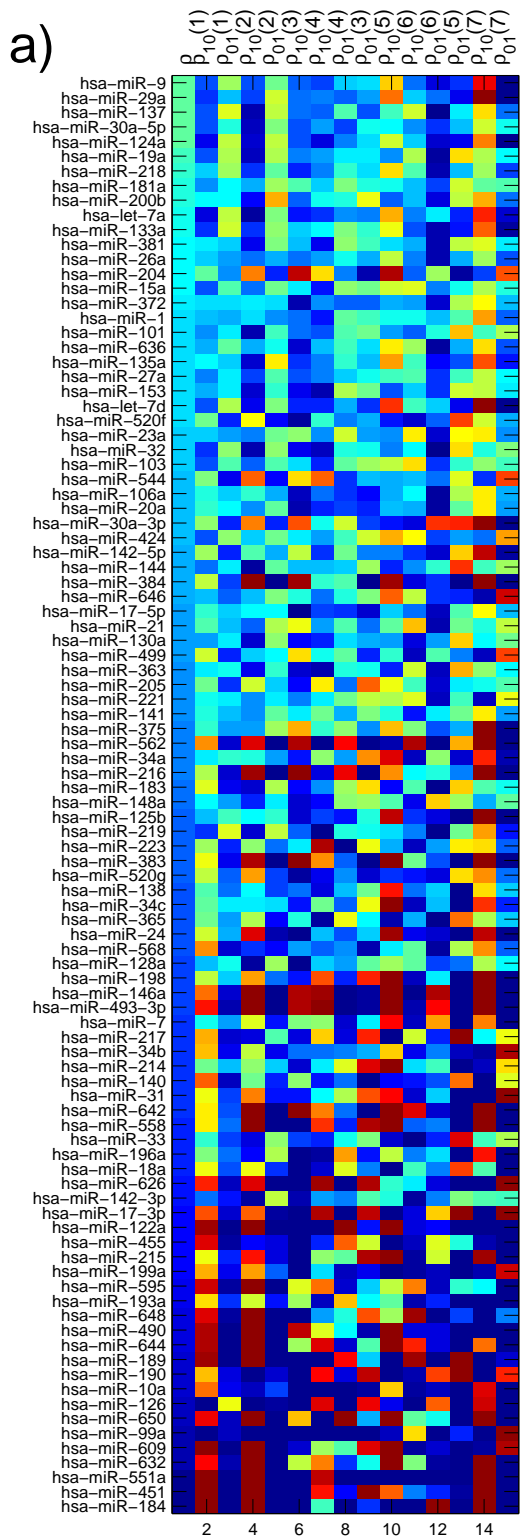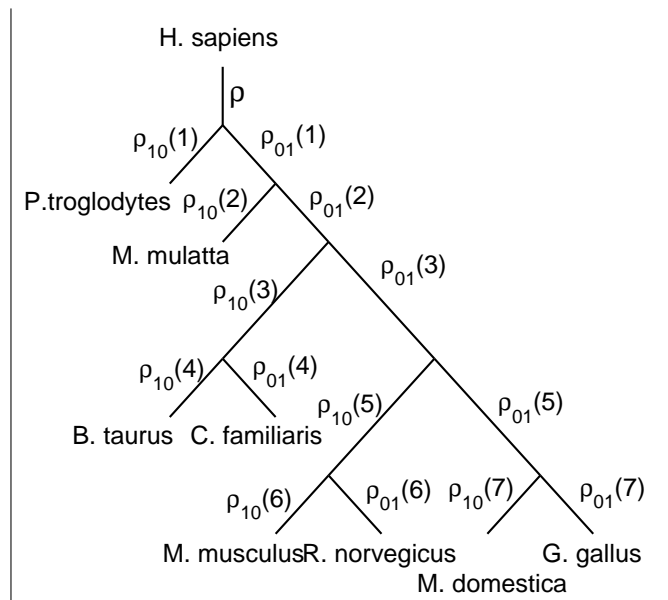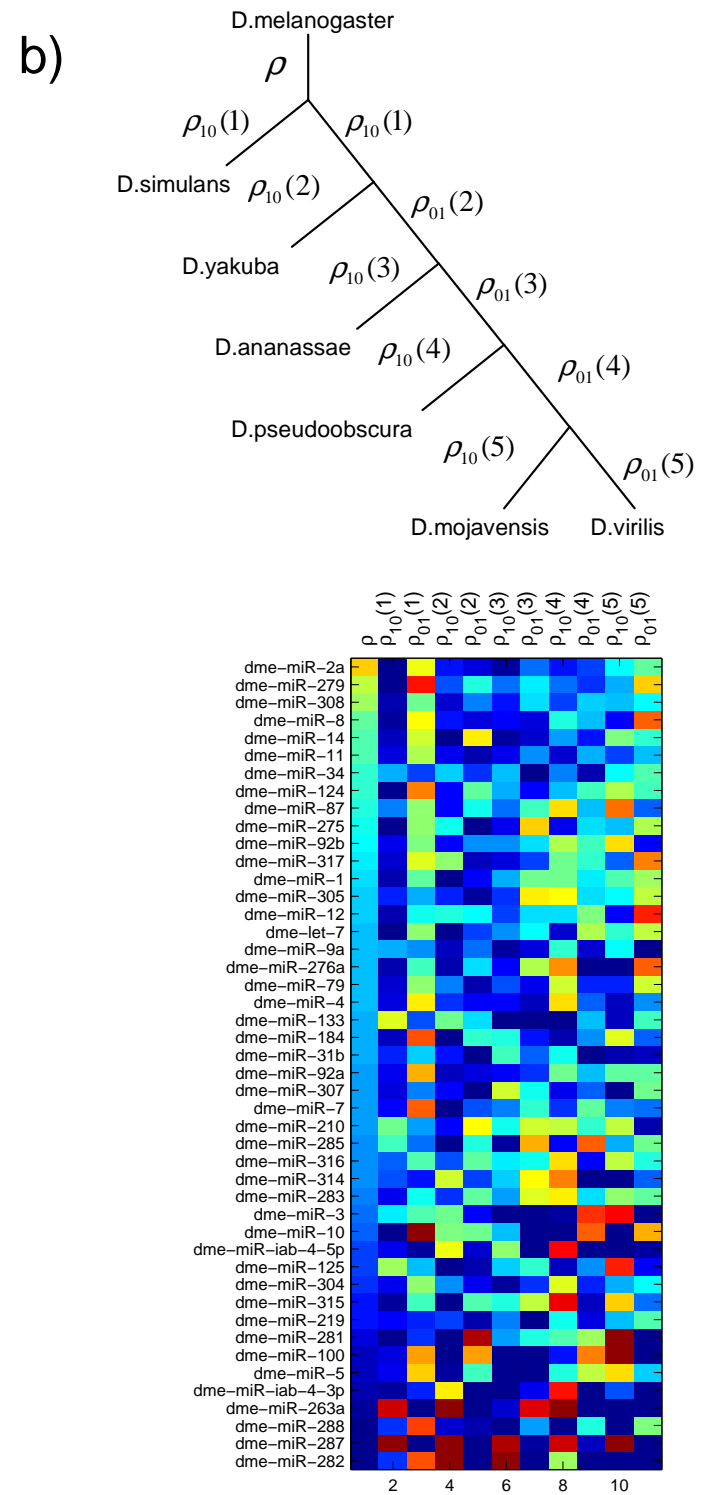

Supplement: Additional File 1 — Phylogenetic distribution of functional target sites. Inferred selection pattern distributions p(s→ MathType@MTEF@5@5@+=feaafiart1ev1aaatCvAUfKttLearuWrP9MDH5MBPbIqV92AaeXatLxBI9gBaebbnrfifHhDYfgasaacH8akY=wiFfYdH8Gipec8Eeeu0xXdbba9frFj0=OqFfea0dXdd9vqai=hGuQ8kuc9pgc9s8qqaq=dirpe0xb9q8qiLsFr0=vr0=vr0dc8meaabaqaciaacaGaaeqabaqabeGadaaakeaacuWGZbWCgaWcaaaa@2E2D@) for all miRNAs that are conserved in all vertebrate (panel a) and all fly (panel b) species. Each row corresponds to a miRNA seed and each column corresponds to one of the variables ρω(k) – where k indicates the internal node in the tree and ω indicates which of the subtrees are under selection – that parametrize p(s→ MathType@MTEF@5@5@+=feaafiart1ev1aaatCvAUfKttLearuWrP9MDH5MBPbIqV92AaeXatLxBI9gBaebbnrfifHhDYfgasaacH8akY=wiFfYdH8Gipec8Eeeu0xXdbba9frFj0=OqFfea0dXdd9vqai=hGuQ8kuc9pgc9s8qqaq=dirpe0xb9q8qiLsFr0=vr0=vr0dc8meaabaqaciaacaGaaeqabaqabeGadaaakeaacuWGZbWCgaWcaaaa@2E2D@) (see Methods). The miRNAs are sorted by the inferred total fraction ρ of putative target sites that is under selection in at least one other species. [file 1471-2105-8-69-S1.pdf]

3 UTR lengths &lt;2000nt

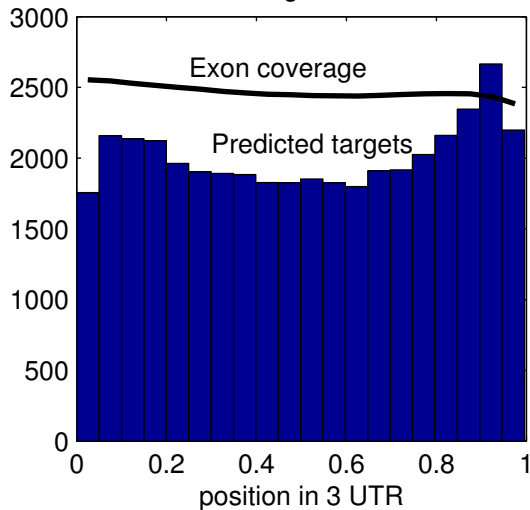

3 UTR lengths &gt;4000nt

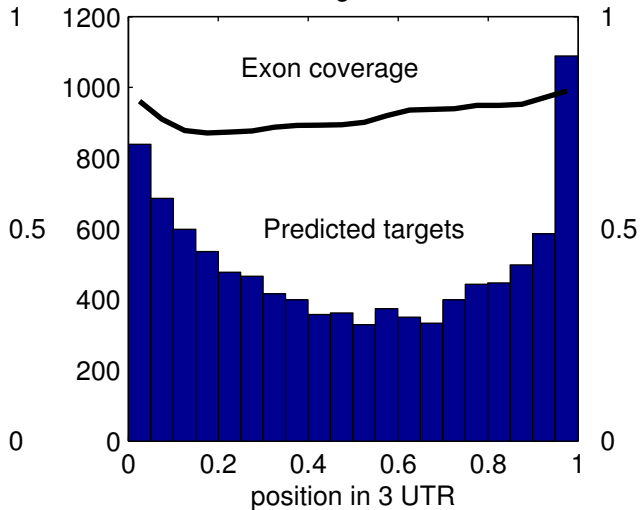

Supplement: Additional File 4 — Profile of the exon coverage of short (left panel) and long (right panel) 3' UTRs. We used the mappings of spliced ESTs from the UCSC database to determine, for each nucleotide in a 3' UTR in our data set, the fraction of times the nucleotide has been observed in an exon, as opposed to an intron. We only used ESTs that mapped uniquely with at least 95% identity to the genome. Genome gaps longer than 30 nucleotides were considered to be introns. The profiles of the computed exon coverage along relatively short (less than 2 kb, left panel) and relatively long (longer than 4 kb, right panel) 3' UTRs are shown in the plots with a continuous line. Also shown are the histograms of the relative positions of predicted sites (with posterior probability ≥ 0.5) in the same 3' UTRs. [file 1471-2105-8-69-S4.pdf]

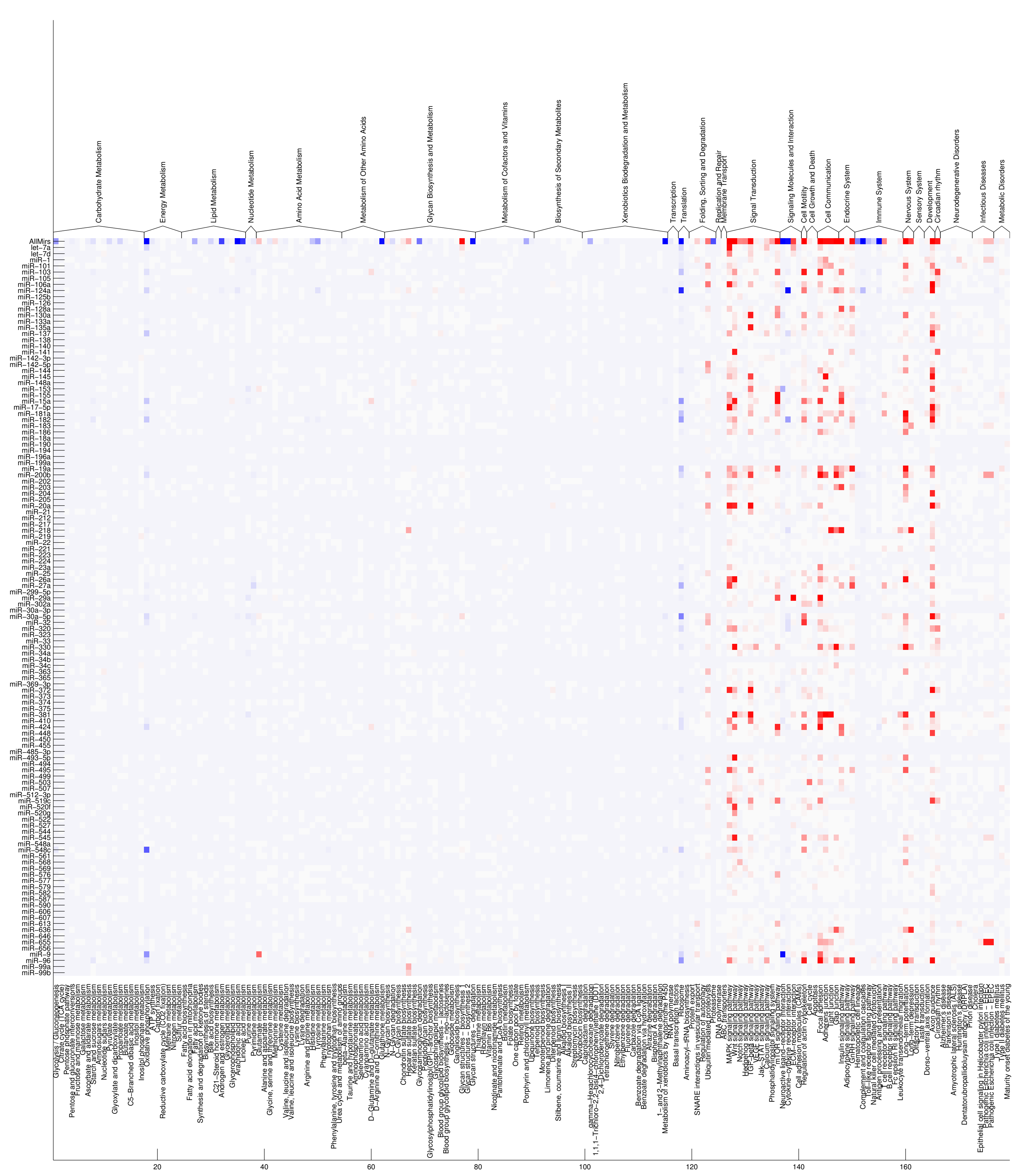

Supplement: Additional File 5 — Pathway analysis for all miRNAs and all KEGG pathways. Representation of individual pathways among the predicted targets of a given miRNA. Each column corresponds to a KEGG pathway and each row to a group of miRNAs with the same seed sequence. Red indicates overrepresentation of the targets of a specific miRNA among the genes in the corresponding pathway, whereas blue indicates depletion. The intensity of the color indicates the posterior probability of the dependent model. Pathways have been grouped in larger functional categories according to the KEGG annotation. [file 1471-2105-8-69-S5.pdf]
